# Therapeutic peptides and proteins: Status and developments in drug delivery

**DOI:** 10.1016/j.jconrel.2026.114895

**Published:** 2026-04-05

**Authors:** Braxten D. Hornsby, Cheng Hua Lee, Cassidy A. Steele, Julius K.K. Tuekpe, Carol S. Lim

**Affiliations:** Department of Molecular Pharmaceutics, University of Utah, Salt Lake City, UT 84112, United. States

**Keywords:** Peptide therapeutics, Protein therapeutics, Molecular engineering, Drug carriers, Formulation strategies, Routes of administration

## Abstract

Peptides and proteins are the most dynamic class of biomolecules, performing a diverse range of physiological functions including biochemical catalysis, ion transport, molecular signaling, and genetic/epigenetic regulation and expression. Considering their indispensable functionality and tremendous diversity, peptide and protein related diseases present both an enormous challenge to human health and a valuable opportunity for therapeutic development. Unfortunately, peptide and protein therapeutics are susceptible to proteolytic degradation and often exhibit poor membrane permeability. Moreover, renal clearance, short circulation time, low plasma stability, and immunogenicity are persistent challenges for delivery. Due to inherent lability in the GI tract and poor absorption and permeability through membranes, the majority of FDA-approved peptide and protein therapeutics are approved for parenteral delivery. However, molecular engineering, drug carriers, and/or co-formulations with effective agents may be used to enhance drug delivery and enable use of more attractive administration routes. This review catalogues and describes established and emerging strategies for chemical and structural modification, formulation, and administration of peptide and protein therapeutics, additionally analyzing how such strategies and technologies have influenced scientifically ground-breaking and commercially successful therapies in the modern market.

## 1. Introduction

Peptides and proteins comprise the most dynamic class of biomolecules, performing a diverse range of physiological functions including biochemical catalysis, ion transport, molecular signaling, and both genetic and epigenetic regulation and expression [1–3]. Moreover, proteins may function as extra- and intra-cellular scaffolds, membrane channels, and receptors [2], while peptides may function as hormones, ion-channel ligands, and anti-infectives [3,4]. The complete human proteome and peptidome are coded within an estimated 20,000–25,000 genes [5,6], with dysregulation, misexpression, and mutation of these genes associated with myriad diseases. Given the profound biological centrality of peptides and proteins and the myriad diseases that may arise from their dysregulation, it is unsurprising that these molecules have become a major focus of modern drug development.

Peptide and protein therapeutics offer several key advantages over traditional small-molecule drugs. First, their larger size and complex three-dimensional structures allow them to engage targets with high specificity and affinity, often binding to shallow or extended protein–protein interaction surfaces that are considered “undruggable” by small molecules. This selectivity typically translates into fewer off-target side effects and a wider therapeutic window. Additionally, many peptides and proteins are derived from or mimic endogenous hormones, cytokines, or growth factors, allowing them to leverage natural signaling pathways with high potency and predictable biological activity. Despite these strengths, peptide and protein drugs still face significant challenges. Their inherent susceptibility to proteolytic degradation and poor membrane permeability limit oral bioavailability, typically necessitating parenteral administration, which can reduce patient compliance. Moreover, renal clearance, short circulation time, low plasma stability, membrane permeability, and immunogenicity remain persistent challenges to the development of new peptide and protein therapeutics [7].

Although numerous reviews have examined peptide and protein drug delivery, the existing literature remains largely fragmented—often focusing narrowly on a single route of administration (such as oral, transdermal, and intranasal) or broadly cataloguing chemical and formulation strategies without integrating them within a modern clinical and commercial context. Moreover, many route-specific analyses are now more than five to ten years out of date, preceding the recent acceleration in entero-pancreatic hormone therapeutics, crystallizable fragment (Fc)-engineered biologics, peptide–drug conjugates, lipid nanoparticle technologies, and device-enabled delivery systems. As a result, the field lacks a contemporary, unified assessment that simultaneously evaluates market status, molecular engineering strategies, drug carrier platforms, and routes of administration in relation to the therapies that have most meaningfully advanced clinical translation.

This review seeks to address that gap by cataloguing and describing the major molecular engineering strategies and drug carrier platforms, both established and emerging, that have contributed toward the apparent momentum of the modern peptide and protein therapeutic market. We also assess the state of these therapies, both those approved by the U.S. Food and Drug Administration (FDA) and those most promising in the clinic, by individual routes of administration, highlighting the most commercially and scientifically successful therapies and the key technologies that have contributed to their success. Important features of highlighted drugs are discussed relative to both their approved route of administration and their intended target. Toward this end, we aim to provide not only a collection of strategies, technologies, and successful therapies in the field of peptide and protein delivery, but also a nuanced analysis of their impact upon the modern market and the trajectory of peptide and protein delivery research.

## 2. Market status

**Fig. 1.**
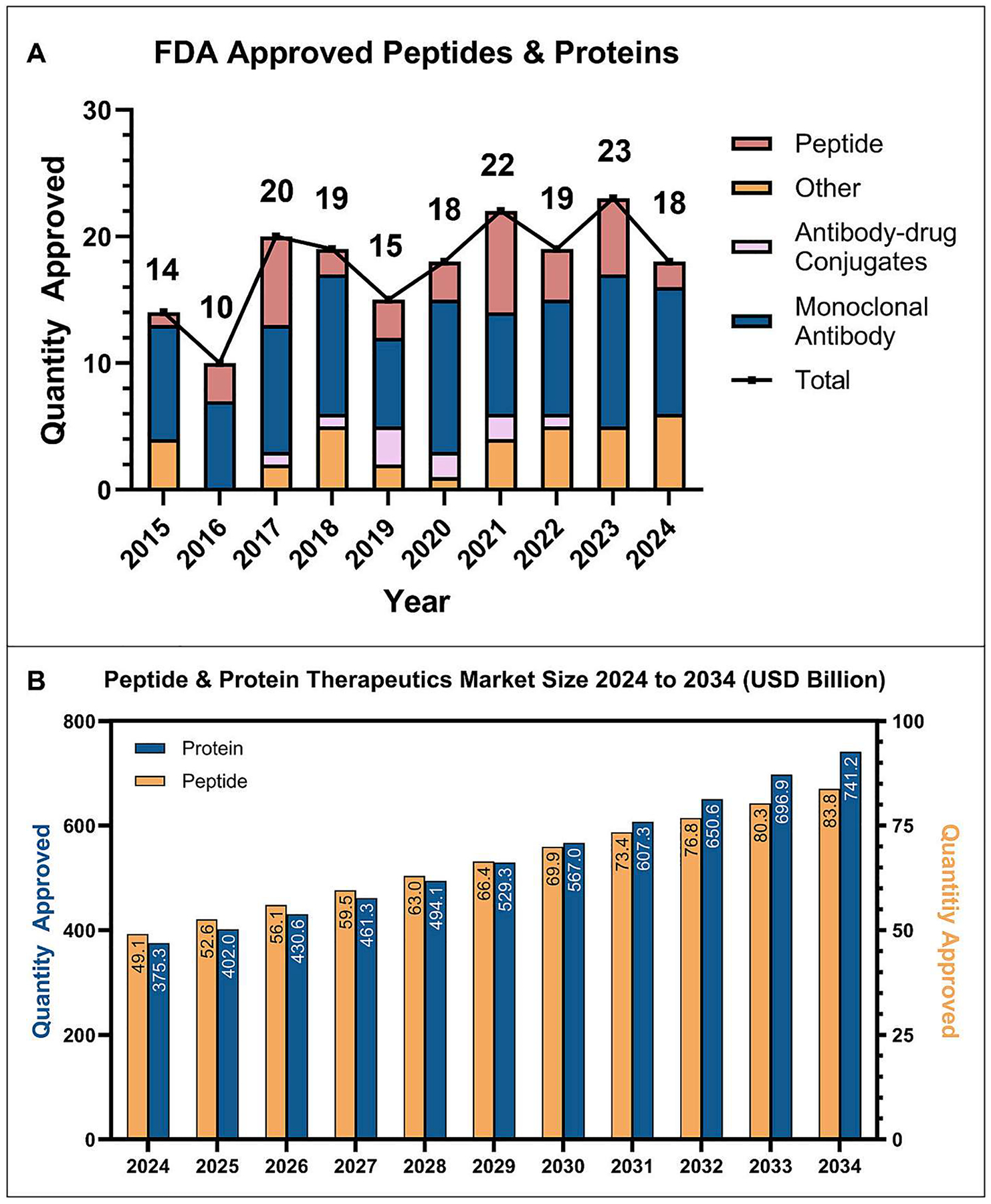
Peptide and protein therapeutics market. (A) The quantity of U.S. Food and Drug Administration (FDA)-approved peptide and protein therapeutics by year are shown for the past decade. (B) The peptide and protein therapeutic market in 2024, accompanied by a ten-year forecast [13,14]. The y-axis on the left represents values for proteins (blue), and the y-axis on the right represents peptides (orange).

More than 200 unique therapeutic peptides and proteins have been approved for clinical use in the United States by the FDA [8], with 178 approved within the last 10 years [9–11] (Fig. 1A) and hundreds more in active development, preclinical, and clinical trials [3,12]. Since the introduction of insulin for the treatment of diabetes mellitus in 1922, global peptide and protein therapeutic markets have grown to approximately $49 billion (USD) and $375 billion respectively and are forecast to attain a combined market value of $825 billion by 2034 (Fig. 1B) [13,14]. The peptide and protein therapeutics market represents 22.5% of the total global pharmaceutical market ($1.67 trillion), notably fortified by the momentum that monoclonal antibodies and entero-pancreatic hormone-based peptides have generated in the last few years [15]. Indeed, of the top ten peptide and protein therapeutics of 2024 by global sales, monoclonal antibodies constitute seven, glucagon-like peptide-1 (GLP-1) receptor agonist (RA) semaglutide (Ozempic^®^) constitutes one, and GLP-1RA/gastric inhibitory polypeptide (GIP)-RA tirzepatide (Mounjaro^®^) constitutes another one, for a total of 9 out of 10 top sellers (Table 1) [15,16].

**Fig. 2.**
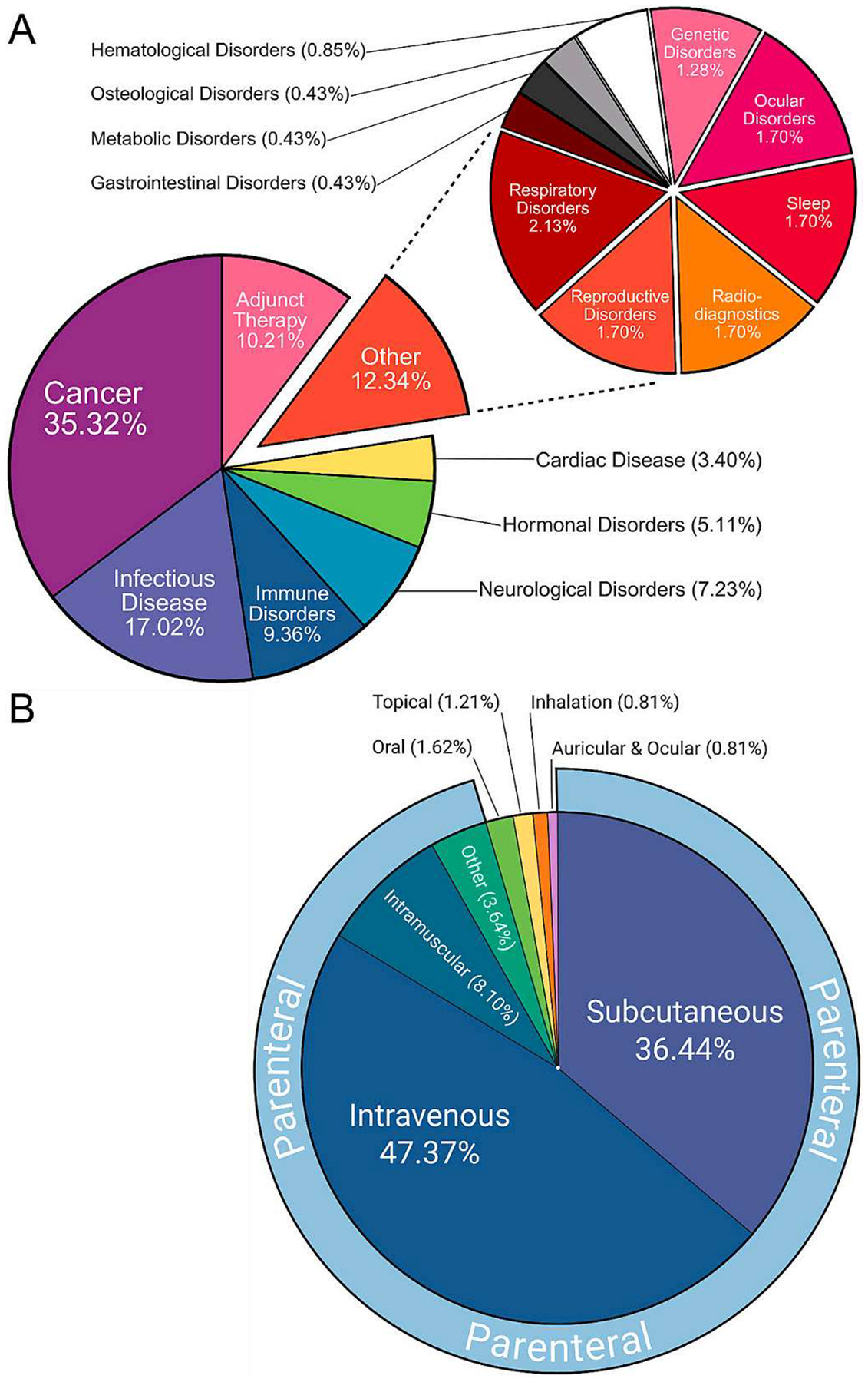
Combined U.S. Food and Drug Administration (FDA)-approved peptide and protein therapeutic markets by: (A) Indication and (B) administration [8, 15]. Created in BioRender. LIM, C. (2025) https://BioRender.com/nlzpzm5.

Cancer is the most common indication (3 of the top 10) among the top selling peptide and protein therapeutics of 2024 (Table 1), in addition to being the most common indication in the total peptide and protein therapeutic market at 35.32% (Fig. 2A) [8,15]. In contrast, only 0.43% of FDA-approved peptide and protein therapeutics are indicated for managing weight loss and metabolic disorders such as type I diabetes, yet semaglutide and tirzepatide dominate the peptide market in sales and rate of growth. Until recently, the peptide and protein market has primarily been influenced by cancer, infectious disease, and immune disorders, with monoclonal antibodies dominating FDA-approved therapies within this space (Fig. 2A) due to their precise targeting, high sensitivity, tunability, and versatile applicability ranging from in-home testing kits to treatment of a wide variety of diseases. However, the recent surge in popularity of entero-pancreatic hormone-based peptides is expected to command greater influence in future markets, and therefore greater investments in research and development. Indeed, new chemical entities (NCEs) retatrutide (triple GLP-1GIP/glucagon RA; TRIUMPH clinical trial) [17], cagrisema (dual GLP-1/amylin RA; REDEFINE clinical trial) [18], and orforglipron (oral GLP-1 RA with no food/water restrictions; ACHIEVE clinical trial) [19,20] have recently begun Phase 3 clinical trials aiming to introduce new target combinations or improve upon available formulation strategies. Moreover, semaglutide and tirzepatide are under active study for expanded indications, with semaglutide exhibiting improved liver histological results in treatment of metabolic dysfunction-associated steatohepatitis (MASH) in part 1 of the ongoing ESSENCE trial [21,22], and tirzepatide exhibiting a risk reduction of worsening heart failure events in certain patients vs placebo in the SUMMIT trial [23]. With a clinical pipeline poised to introduce new receptor targets and target combinations, improve upon oral formulation strategies, and expand approved treatment indications for already approved therapies, growing interest in entero-pancreatic hormone-based peptides is evident and abundant.

The recent dominance of GLP-1 receptor agonists exemplifies how market expansion depends on coordinated advances in delivery engineering. Native GLP-1 is rapidly degraded by dipeptidyl peptidase IV (DPP-IV) and cleared renally [24–27], yet clinically successful analogs incorporate amino acid substitutions to resist enzymatic cleavage and fatty-acid lipidation to promote albumin binding, enabling once-daily or once-weekly dosing [28–32]. The subsequent approval of oral semaglutide required further formulation innovation, specifically co-formulation with the absorption enhancer salcaprozate sodium (SNAC) to enable gastric uptake under acidic conditions [33–36]. Thus, commercial success in metabolic disease reflects integration of molecular stabilization, half-life extension, and absorption-enhancing technologies rather than target biology alone.

More broadly, many top-selling peptide and protein therapeutics derive their clinical and market durability from platform-level delivery innovations. Fc engineering and glyco-optimization extend antibody half-life through FcRn recycling [37–40], while recombinant human hyaluronidase has enabled transition of several biologics from intravenous to subcutaneous administration, improving convenience and expanding access [41]. In parallel, regulatory validation of lipid nanoparticle systems and clinically established hydrogel and nanoparticle platforms signal that carrier technologies increasingly function as enabling infrastructures for biologic development [42–51]. Collectively, these trends indicate that the trajectory of the peptide and protein market is inseparable from advances in delivery science, which transform inherently labile biomolecules into durable and commercially viable therapies.

## 3. Molecular engineering strategies to improve drug delivery

### 3.1. Overview

**Fig. 3.**
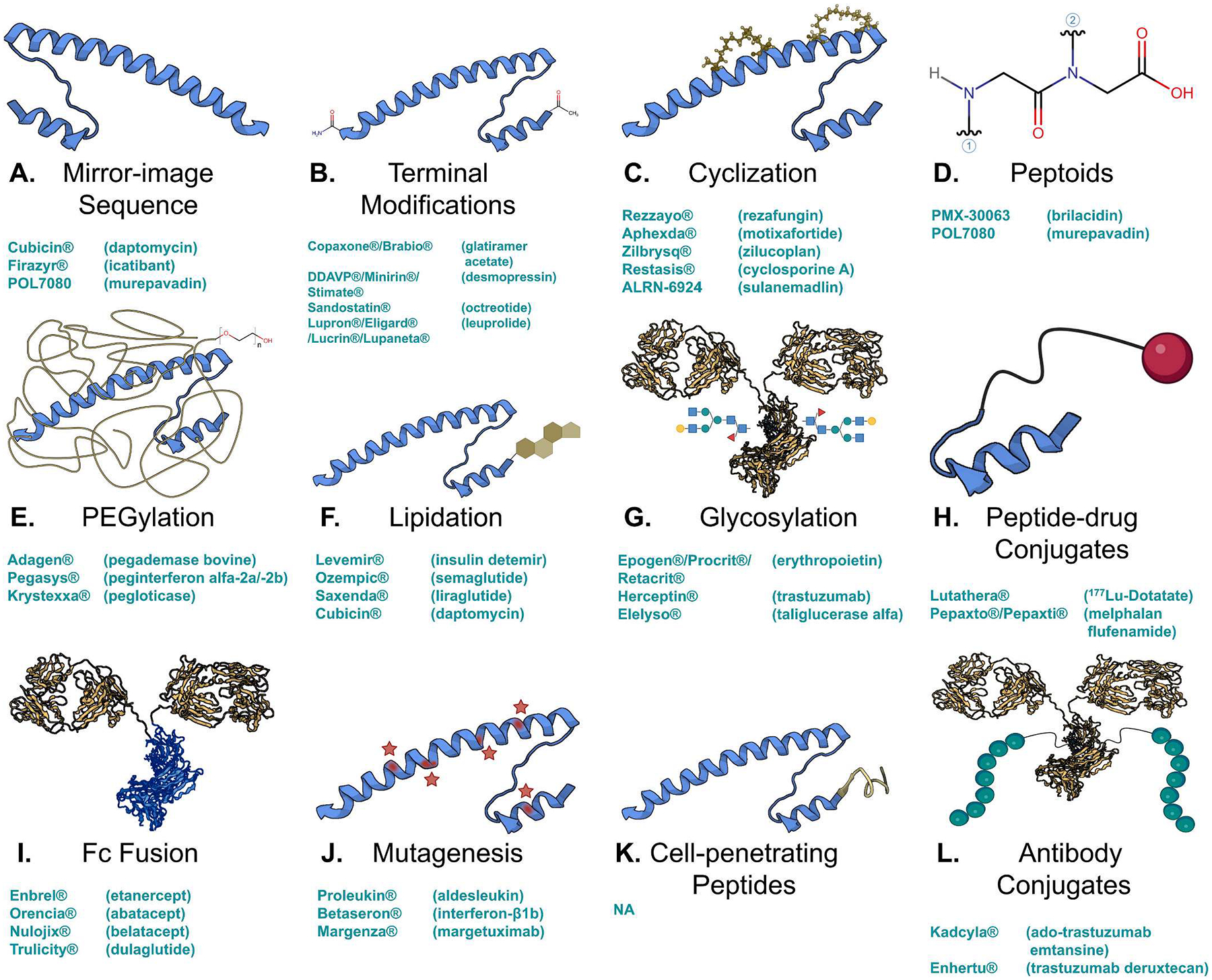
Predominant chemical and structural modification strategies for FDA-approved and investigational peptide and protein therapeutics. (A) Mirror-image protein composed of D-amino acids. (B) N-terminal acetylation and C-terminal amidation. (C) Hydrocarbon stapled peptide, representing one of many peptide cyclization strategies. (D) Schematic representation of a peptoid. (E) PEGylated protein with an enlarged hydrodynamic radius. (F) N-terminal cholesterol conjugation. (G) Glycosylation of IgG2a monoclonal antibody. (H) C-terminal linkage of a drug payload to a peptide targeting moiety. (I) Fragment crystallizable region (Fc) fusion. (J) Mutagenesis, achievable by either targeted mutagenesis (rational design) or random mutagenesis (directed evolution). (K) N-terminal, linear cell-penetrating peptide (CPP). (L) Antibody-drug conjugate (ADC) using a peptide payload. Representative drugs for each class (when available) are in teal text. Created in BioRender. LIM, C. (2025) https://BioRender.com/5lbm6gb.

The biological activity of peptide and protein therapeutics is dependent upon their chemical structure, conformational dynamics, and physicochemical properties. Despite their specificity and potency, these biomolecules are often hindered by intrinsic limitations such as rapid proteolytic degradation, aggregation, poor solubility, and short plasma half-life. To function effectively as drugs, peptides and proteins must be chemically and structurally optimized to achieve stability under both physiological and storage conditions, resist enzymatic cleavage, and maintain favorable pharmacokinetic and pharmacodynamic behavior. As part of or following initial synthesis or recombinant expression, engineering techniques are employed to mimic or stabilize ideal secondary structures, enhance receptor selectivity, and fine-tune solubility and bioavailability. These modifications may be performed through a variety of strategies (Fig. 3).

### 3.2. Strategies to improve stability and bioactivity

#### 3.2.1. Backbone and side-chain modifications

Backbone modifications may be used to mitigate proteolytic degradation through strategies such as D-amino acid substitution (Fig. 3A), *N*-methylation, and targeted residue replacement [52–54]. Moreover, advances in synthetic chemistry and computational modeling have expanded backbone engineering beyond protease resistance to allow tuning of drug solubility, permeability, and binding selectivity [54,55]. Approved drugs illustrate these principles: teduglutide substitutes Ala→Gly at position 2 to block DPP-IV and extend half-life [24], semaglutide includes Ala8 → Aib (α-aminoisobutyric acid) and Lys34 Arg → mutations boost metabolic stability [56–58], cyclosporine A employs *N*-methylated residues to restrict conformational flexibility and facilitate oral absorption [59], and the antimicrobial lipopeptide daptomycin incorporates D-amino acids and nonproteinogenic units to improve structural rigidity and protease resistance [60].

Side-chain substitutions may be used to enhance stability, solubility, and target affinity through analog replacement or post-synthetic edits, typically at Lys/Cys/Tyr residues. Many entero-pancreatic hormone-based peptides are characterized by side-chain modifications, such as semaglutide which includes a 2-aminoisobutyric acid substitution to reduce degradation, as well as a C18 fatty acid conjugation to a lysine side-chain to enhance albumin binding and reduce renal clearance [57]. Additionally, side-chain modifications are essential for many targeted delivery systems such as antibody–drug conjugates (ADCs), where drugs like trastuzumab emtansine (Kadcyla^®^) [61] and brentuximab vedotin (Adcetris^®^) [62] exploit lysine- or cysteine-linked conjugation to deliver cytotoxic agents selectively to cancer cells.

#### 3.2.2. N- and C-terminus modifications

Peptides and proteins are highly susceptible to exopeptidase cleavage at their termini. N- and C-terminal modifications enhance proteolytic resistance, extend half-life, and improve bioavailability (Fig. 3B) [63]. The most common approaches, *N*-acetylation and C-amidation, neutralize terminal charges to mimic native proteins, improving intracellular delivery and reducing enzyme recognition [64]. Alternatively, a variety of terminal conjugation techniques have proven effective in improving circulation time and reducing proteolysis, often by promoting albumin adsorption, as demonstrated by Insulin degludec (Tresiba^®^) which is myristoylated at LysB29 to facilitate albumin binding [65], or albiglutide (Tanzeum^®^/Eperzan^®^) which is fused to human albumin at the C-terminus [66].

#### 3.2.3. Cyclization

Cyclic peptides improve stability, binding affinity, and pharmacokinetics relative to linear analogs by eliminating terminal residues, reducing backbone flexibility, and pre-organizing bioactive conformations [67,68]. Cyclization strategies include head-to-tail, heterodetic, and side-chain crosslinks, and can tune permeability and solubility [67,68]. Notably, three of eight FDA peptide approvals in 2023–2024 were cyclic (rezafungin, motixafortide, zilucoplan [9], Orally bioavailable peptides include cyclosporine A and voclosporin (head-to-tail) and desmopressin (1,6-disulfide), and clinically successful heterodetic cyclizations are included in daptomycin and polymyxins [69,70]. Stapled peptides further stabilize α-helices via covalent side-chain linkages, enhancing proteolytic resistance and permeability (Fig. 3C) [71,72]. Sulanemadlin (ALRN-6924) is the stapled peptide to progress the furthest clinically, reaching Phase 1/2 trials before discontinuation [73].

Sulanemadlin is a cell-permeable, stapled α-helical peptide designed to inhibit both mouse double minute 2 homolog (MDM2) and murine double minute X (MDMX), thereby reactivating wild-type p53 signaling in tumors retaining functional TP53 [73,74]. In early-phase clinical studies, it demonstrated pharmacodynamic evidence of p53 pathway activation, including induction of canonical transcriptional targets; however, objective response rates in advanced solid tumors and hematologic malignancies were modest and predominantly cytostatic rather than tumor-regressive [73]. As observed with other agents targeting the MDM2–p53 axis, on-target hematologic toxicities, including thrombocytopenia, neutropenia, and anemia, limited dose intensity and narrowed the therapeutic window, reflecting the essential role of p53 signaling in normal hematopoietic progenitors [75,76]. The broader clinical experience with MDM2 inhibitors, including idasanutlin and milademetan, has similarly revealed a recurring efficacy–toxicity tradeoff that has impeded late-stage success in oncology [75,76]. In this context, sulanemadlin lacked sufficient clinical differentiation to justify continued development in advanced cancer.

#### 3.2.4. Peptoids

Peptoids are N-substituted glycines in which side chains are attached to the amide nitrogen rather than the α-carbon, yielding an achiral backbone with high conformational flexibility and strong proteolytic resistance due to reduced amide recognition (Fig. 3D) [77]. Pharmacophore geometry can be stabilized through bulky or chiral side chains, π-stacking designs, and cyclization to bias preferred rotamers [78,79]. Beyond stability, peptoids often exhibit improved permeability, intracellular delivery, and lower immunogenicity than peptides [80,81]. Preclinically, antimicrobial peptoids (“ampetoids”) demonstrate low-micromolar activity against multidrug-resistant (MDR) Gram-negative bacteria [77,82]. Despite strong preclinical rationale, high-profile clinical programs have failed or stalled due to toxicity, insufficient efficacy in complex infections, or strategic discontinuation. Two instructive examples are brilacidin (PMX-30063) [83] and murepavadin (POL7080) [84].

Brilacidin is a non-peptidic arylamide foldamer designed to mimic host-defense peptides through amphipathic, membrane-targeting activity, producing rapid bactericidal effects via preferential interaction with negatively charged bacterial phospholipids and membrane depolarization [85]. Although Phase 2 studies in acute bacterial skin and skin structure infections demonstrated promising efficacy, broader systemic development did not advance, reflecting persistent concerns common to membrane-active agents, including a narrow therapeutic window, incomplete selectivity between bacterial and host membranes, and pharmacokinetic limitations in achieving adequate exposure at deep infection sites without dose-limiting toxicity [86]. In contrast, murepavadin represents a pathogen-specific cyclic β-hairpin peptidomimetic that targets the essential outer membrane protein lipopolysaccharide transport protein D (LptD) in *Pseudomonas aeruginosa*, disrupting lipopolysaccharide transport and outer membrane biogenesis [87]. Despite encouraging early-phase data and progression into Phase 3 trials for nosocomial pneumonia, intravenous development was halted following an unexpected signal of acute kidney injury, likely related to systemic exposure levels required for pulmonary infections and compounded by altered pharmacokinetics in critically ill patients [88,89].

#### 3.2.5. Comparative analysis of stability and bioactivity strategies

While backbone modification, terminal modification, cyclization, and peptoid substitution all enhance proteolytic stability, they do so through mechanistically distinct structural principles (Table 2). Backbone engineering primarily disrupts protease recognition motifs and increases conformational rigidity without necessarily eliminating linear topology. In contrast, cyclization removes free termini entirely and pre-organizes the peptide into a constrained bioactive conformation, often simultaneously enhancing receptor affinity and metabolic stability. Terminal modifications are comparatively minimalistic interventions. They provide protection from exopeptidases but generally exert modest effects on higher-order structure unless coupled with conjugation strategies like lipidation of Fc fusion. Peptoids represent the most structurally divergent approach, eliminating backbone chirality and shifting side chains to the amide nitrogen—fundamentally altering hydrogen-bonding patterns and protease recognition but potentially reducing predictable secondary structure. A key distinction among these approaches lies in structure–activity preservation. Cyclization and rational backbone substitutions often maintain pharmacophore geometry, whereas extensive peptoid substitution may require re-optimization of binding interactions. Thus, the more radical the structural modification, the greater the need for re-validation of potency. Importantly, these methods are not mutually exclusive. Modern peptide drugs frequently combine amino acid substitution, lipidation, and conformational constraint to achieve synergistic improvements in stability and receptor selectivity.

### 3.3. Strategies to improve pharmacokinetics (PK) and pharmacodynamics (PD)

#### 3.3.1. PEGylation

Polyethylene glycol conjugation (PEGylation) is a widely used and clinically validated strategy to enhance biological drug delivery (Fig. 3E). Hydrophilic PEG chains increase hydrodynamic size and molecular weight, reducing glomerular filtration and prolonging systemic circulation [90]. PEGylation also shields drugs from proteolytic degradation and immune recognition, improving metabolic stability and reducing immunogenicity [90]. Since FDA approval of pegademase bovine (Adagen^®^) for severe combined immunodeficiency in 1990, multiple PEGylated therapeutics have followed, including peginterferon alfa-2a/–2b (Pegasys^®^, PegIntron^®^) for hepatitis [65] and pegloticase (Krystexxa^®^) for gout [91], providing extended half-life and reduced dosing frequency.

Early PEGylation approaches relied on nonspecific lysine or N-terminal attachment, producing heterogeneous conjugates with variable activity [92]. Advances in site-specific PEGylation using engineered cysteines, unnatural amino acids, and genetic code expansion now enable precise, uniform conjugation, exemplified by certolizumab pegol (Cimzia^®^) and turoctocog alfa pegol (Esperoct^®^) [93]. However, bulky PEG chains can impair receptor binding and reduce bioactivity, particularly near binding interfaces or at high PEG density [92], Additionally, anti-PEG antibodies may accelerate clearance, and non-biodegradable PEG accumulation raises long-term safety concerns [94]. These limitations have driven interest in biodegradable linkers and alternative hydrophilic polymers such as poly(2-oxazoline) and polysarcosine [95,96].

#### 3.3.2. Lipidation

Lipid conjugation (lipidation) enhances peptide and protein pharmacokinetics by promoting reversible binding to human serum albumin to reduce renal clearance and proteolysis [97]. Additionally, increased hydrophobicity can prolong circulation and improve cellular uptake [98–100]. Lipid placement must be optimized to preserve activity and avoid aggregation or immunogenicity. Cholesterol conjugation represents a related strategy, anchoring peptides to membranes or engaging low-density lipoprotein (LDL) receptors to increase local concentration and uptake (Fig. 3F) [101,102]. Clinically, lipidation is well exemplified in entero-pancreatic therapies—including insulin detemir (C14 myristate) and GLP-1 agonists such as liraglutide (C16), semaglutide (C18 diacid), and tirzepatide (branched C20), enabling daily-to-weekly dosing [56]. The cyclic lipopeptide daptomycin also illustrates lipidation-driven antimicrobial efficacy [69].

#### 3.3.3. Glycosylation/Glycoengineering

Glyeosylation is a co- or post-translational modification in which carbohydrates are covalently attached to asparagine (N-linked) or serine/threonine (O-linked) residues by intracellular glycosyltransferases (Fig. 3G) [103]. Glycan branching and sialylation generate glycoform diversity that influences protein folding, stability, pharmacokinetics, and immunogenicity [104]. Modern biotherapeutic design exploits recombinant glycoengineering by introducing or deleting N-lycosylation motifs, remodeling glycans post-expression, or engineering host-cell pathways to produce humanized, homogeneous glycoforms with improved half-life or effector function [37]. Terminal sialylation extends circulation, while Fc afucosylation enhances antibody-dependent cellular cytotoxicity [37,38]. Many FDA-approved proteins are glycosylated [37,38], including erythropoietin, which requires glycans for receptor binding and persistence [105], therapeutic antibodies such as trastuzumab that depend on Fc glycans for antibody-dependent cellular cytotoxicity (ADCC) 106], and enzymes like taliglucerase alfa that use mannose-based targeting for cellular uptake [105].

#### 3.3.4. Peptide-drug conjugates (PDCs)/prodrugs

Peptide–drug conjugates (PDCs) are targeted, modular prodrugs that link cytotoxins, radionuclides, or biologics to short biodegradable peptides via cleavable linkers (Fig. 3H) [107,108]. The peptide moiety enables control over receptor specificity, solubility, and pharmacokinetics while maintaining low immunogenicity. PDCs remain inactive until triggered by tumor microenvironment cues such as enzymatic cleavage, pH shifts, or redox gradients, releasing their payload locally and minimizing systemic toxicity [109,110]. Their small size enhances tissue penetration compared with antibody–drug conjugates, and albumin-binding or lipid motifs can prolong circulation [107,110]. Clinically, PDCs have been approved for multiple modalities. ^177^L-DOTATATE (Lutathera^®^) targets somatostatin receptor 2 for localized radiotherapy in neuroendocrine tumors [111], while melphalan flufenamide (Pepaxto^®^) employs aminopeptidase-sensitive linkers for selective activation in multiple myeloma [112], an approach pioneered by early cathepsin B and legumain-cleavable peptide–methotrexate conjugates [113].

#### 3.3.5. Protein fusions

Protein fusions extend circulation time by linking therapeutics to IgG Fc or human serum albumin (HSA) (Fig. 3I), exploiting neonatal Fc receptor (FcRn) recycling (Fig. 5B) to avoid lysosomal degradation and prolong half-life [39,40]. This strategy has produced multiple FDA-approved drugs, including etanercept (TNFR2–Fc) for rheumatoid arthritis, and abatacept and belatacept (CTLA4–Fc) for autoimmunity and transplantation [114–116]. Fc and albumin fusions also enable long-acting formulations such as dulaglutide (GLP-1–Fc), albiglutide (GLP-1-albumin), and extended coagulation factors Eloctate^®^, Alprolix^®^, and Idelvion^®^ [117,118]. Fusions have also been used to enhance targeting or promote immune redirection, exemplified by denileukin diftitox, romiplostim, and the T-cell–engaging fusion tebentafusp [119–121].

#### 3.3.6. Comparative analysis of pharmacokinetic (PK) and Pharmacodynamic (PD) strategies

Each PK/PD strategy influences drug performance through mechanistically distinct means (Table 3). PEGylation and glycosylation primarily increase hydrodynamic radius, reducing renal clearance and shielding from immune detection. PEGylation offers tunable, predictable half-life extension but may reduce receptor binding and provoke anti-PEG antibodies. Glycosylation, in contrast, is biologically native and can enhance both stability and effector function, such as in Fc-mediated ADCCs, though glycoform heterogeneity complicates manufacturing. Lipidation achieves half-life extension through reversible albumin binding rather than bulk hydrodynamic shielding. Compared with PEGylation, lipidation often better preserves receptor affinity but introduces risks of aggregation or altered biodistribution if hydrophobicity is excessive. Protein fusions (Fc or albumin) provide the most dramatic half-life extension by exploiting FcRn recycling. However, they significantly increase molecular size and complexity, potentially limiting tissue penetration relative to smaller conjugates. Peptide–drug conjugates (PDCs) represent a distinct pharmacodynamic strategy. Rather than merely prolonging exposure, they modulate spatial activation of payloads, enhancing potency and reducing systemic toxicity. Thus, whereas PEGylation and lipidation are PK-dominant strategies, PDCs and Fc engineering alter both PK and PD.

### 3.4. Strategies to improve targeting and potency

#### 3.4.1. Directed evolution and rational design

Peptide and protein engineering enable the design of therapeutics with enhanced potency, selectivity, and stability through two distinct yet complimentary mutagenic (Fig. 3J) strategies: Directed evolution and rational design. Directed evolution mimics natural selection by applying iterative random mutagenesis via error-prone polymerase chain reaction (PCR), DNA shuffling, or saturation mutagenesis followed by screening of large variant libraries [122]. Beneficial mutations accumulate across rounds, often revealing solutions inaccessible to rational prediction [123]. Display technologies such as phage display allow rapid enrichment of high-affinity variants, exemplified by adalimumab (Humira^®^), the first fully human antibody discovered by phage display [124,125]. Directed evolution has also enabled affinity maturation of T-cell receptor–based biologics, enzymes, bispecific antibodies, and antibody–drug conjugates [126–128].

In contrast, rational design applies hypothesis-driven mutagenesis informed by structural and biophysical data to tune folding, stability, or binding specificity. Structural techniques including x-ray crystallography, cryo-electron microscopy (cryo-EM), nuclear magnetic resonance (NMR) spectroscopy, and atomic force spectroscopy (AFM) combined with molecular dynamics simulations enable atomic-level prediction of substitution effects [129]. This approach has produced approved therapeutics such as interferon-β1b which incorporates cysteine substitutions to improve folding, solubility, and reduce aggregation [130], as well as Fc-engineered antibodies like margetuximab with optimized effector function and half-life [131].

Modern workflows increasingly integrate both paradigms—rationally identifying hotspot residues before evolving them empirically [132]. In these workflows, rational modeling first identifies “hotspot” residues or structural regions critical to stability or activity, with directed evolution or focused saturation mutagenesis subsequently applied to refine these features empirically. AI-enabled tools such as AlphaFold2, ProteinMPNN, and DeepSequence further accelerate optimization, reducing experimental screening and enabling improved antibodies, cytokines, and Fc-fusion proteins [133–140].

#### 3.4.2. Cell-penetrating peptides (CPPs) and cell-targeting peptides (CTPs)

Cell-penetrating peptides (CPPs) are short, often cationic peptides that cross cellular membranes to deliver proteins, nucleic acids, or small molecules via direct translocation or endocytic pathways, frequently driven by arginine-rich interactions with anionic membranes (Fig. 3K) [141,142]. The azurin-derived CPP p28 advanced to Phase 1 clinical testing, demonstrating tolerability and p53 pathway engagement, although no CPP–cargo conjugate has been FDA approved [143]. In contrast, receptor-targeting peptides direct therapeutics to specific tissues through high-affinity receptor binding and receptor-mediated endocytosis, as in the FDA-approved radiotherapeutic ^177^Lu-DOTATATE (Lutathera^®^) [46]. Hybrid CPP–targeting designs aim to combine specificity with efficient cytosolic delivery but face challenges including off-target uptake, proteolysis, endosomal trapping, and receptor heterogeneity [141,142].

#### 3.4.3. Antibody conjugates

Antibody–drug conjugates (ADCs) combine monoclonal antibody specificity with cytotoxic drugs for targeted cancer therapy (Fig. 3L). Each ADC includes a tumor-specific antibody, a cytotoxic payload, and a linker that ensures stability in plasma and controlled intracellular release via enzymatic or pH-sensitive cleavage [144]. Early ADCs were limited by immunogenic antibodies, unstable linkers, and weak payloads, but advances in antibody humanization and conjugation chemistry have transformed the field. Ado-trastuzumab emtansine (Kadcyla^®^) was the first HER2-targeted ADC to improve survival in metastatic breast cancer [145]. Its successor, trastuzumab deruxtecan, uses a cleavable tetrapeptide linker and membrane-permeable topoisomerase I inhibitor to achieve high efficacy, even in low-HER2 tumors [146].

Traditional lysine- or cysteine-based coupling yields heterogeneous mixtures with variable drug-to-antibody ratios (DARs), leading to batch variability in stability, efficacy, and toxicity [147–150]. Modern site-specific conjugation technologies, including THIOMAB^™^ cysteine engineering, aldehyde-tag/HIPS ligation, and enzymatic couplings, address this by engineering unique reactive sites or chemical handles [151–156]. Incorporation of unnatural amino acids provides orthogonal handles for site-defined PEGylation or drug linkage [157], while hydrophilic polysarcosine linkers improve solubility and pharmacokinetics [158]. New cleavable linkers (β-glucuronide, protease-sensitive) further improve tumor selectivity, exemplified by the DXd system in DS-8201a [159].

#### 3.4.4. Comparative analysis of targeting and potency strategies

Engineering strategies may be selected depending upon their influence toward specificity, cell penetration, and potency enhancement, but engineering complexity must also be considered (Table 4). Directed evolution and rational design represent complementary paradigms. Directed evolution excels in optimizing affinity and stability when structure-function relationships are incompletely understood, but it is resource-intensive. Rational design is more hypothesis-driven and efficient when high-resolution structural data exist, though it may miss emergent beneficial mutations. CPPs enhance intracellular delivery but lack intrinsic specificity, whereas cell-targeting peptides (CTPs) improve tissue selectivity through receptor-mediated uptake. Antibody–drug conjugates (ADCs) provide the highest degree of targeting specificity but at the cost of molecular size and manufacturing complexity. Thus, targeting strategies trade off specificity, penetration depth, and complexity. Small targeting peptides penetrate tumors more efficiently than antibodies but may bind with lower affinity. ADCs exhibit superior selectivity but may struggle with tumor penetration in dense tissues.

## 4. Drug carrier systems

### 4.1. Overview

**Fig. 4.**
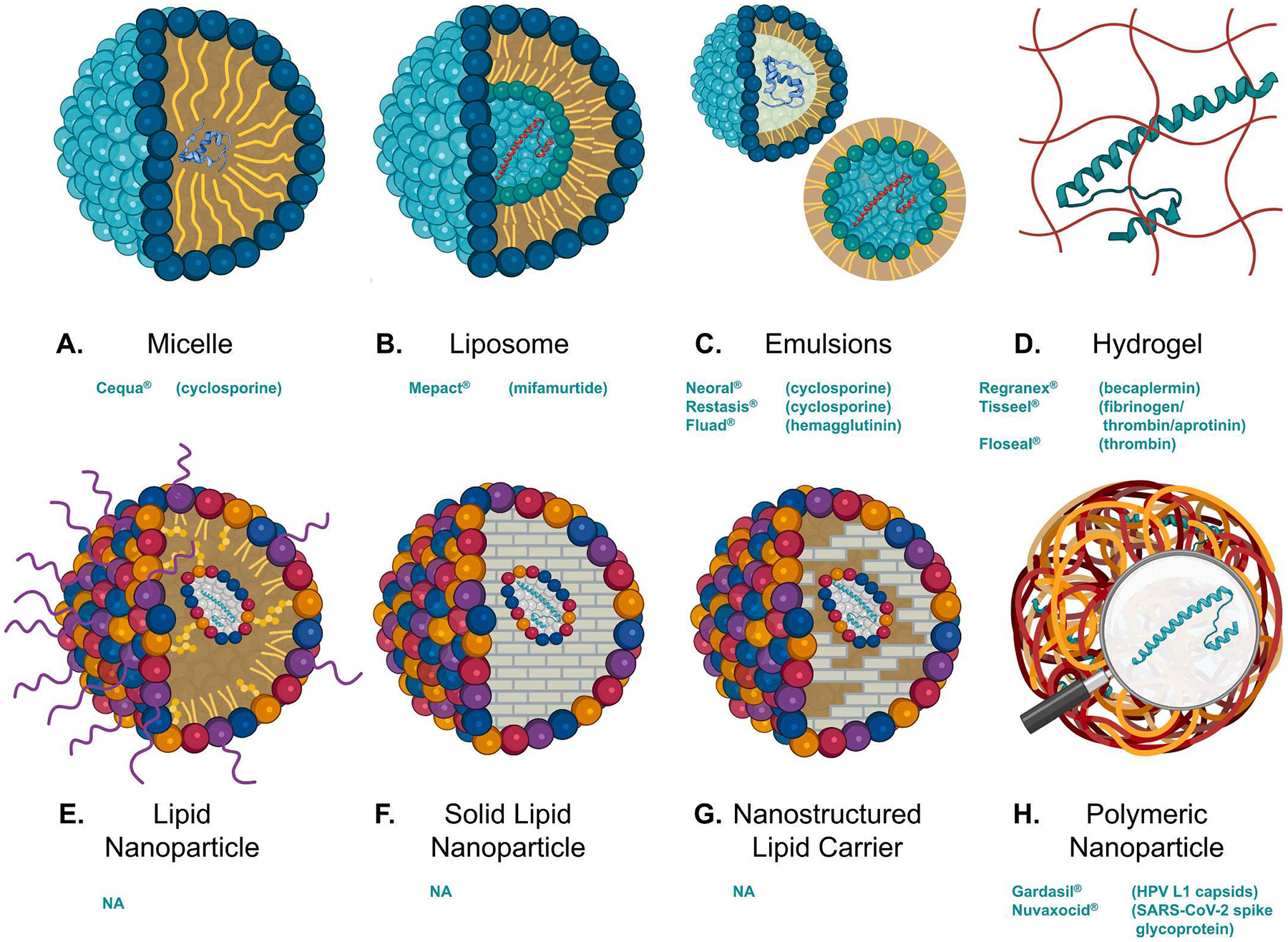
Predominant carrier systems for FDA-approved and investigational peptide and protein therapeutics. Peptide/protein therapy (coil) is shown in aqua or red in the center of each example. (A) Regular micelle with hydrophobic core (orange) for lipophilic drugs. Reverse micelles may also be used for hydrophilic drugs (not shown). (B) Unilamellar liposome with a single lipid bilayer, an aqueous core (aqua) and hydrophobic shell (orange) for delivery of either hydrophilic or lipophilic drugs. (C) water-in-oil (W/O) and oil-in-water (O/W) micro/nanoemulsions for hydrophilic and lipophilic drugs respectively. (D) Hydrogel with physically entrapped protein, although non-covalent and covalent binding may also be used (not shown). (E) Lipid nanoparticle consisting of polyethylene glycol (PEG) conjugated lipids (purple), cholesterol (orange), an ionizable lipid (blue), and a phospholipid (red), with an amorphous lipid core. (F) Solid lipid nanoparticle (SLN) with a structured, solid lipid core. (G) Nanostructured lipid carrier (NLC) with a mixed solid and amorphous lipid core. (H) Polymeric nanoparticle with an entrapped protein, although proteins may also be encapsulated through chemical bonding. Representative drugs for each class (when available) are in teal text. Created in BioRender. LIM, C. (2025) https://BioRender.com/mrwd2db.

The clinical expansion of peptide and protein therapeutics has intensified the need for delivery systems capable of preserving structural integrity while overcoming biological barriers [42–51]. Although advances in protein engineering and chemical modification have extended half-life and improved pharmacokinetics for certain biologics, many therapeutic candidates still depend on carrier systems to achieve effective biodistribution, intracellular access, or sustained release [42–51]. As a result, drug carrier technologies have become central to unlocking the full therapeutic potential of macromolecular medicines (Fig. 4).

Despite decades of research and substantial clinical success in small-molecule and, more recently, nucleic acid delivery, translation of nanocarriers for peptides and proteins remains comparatively limited [160–168], This gap reflects fundamental physicochemical differences between folded proteins and other cargo classes. Proteins possess defined tertiary and quaternary structures that are sensitive to interfacial stress, shear forces, pH shifts, organic solvents, and dehydration during formulation [160–169]. Their relatively large hydrodynamic size and heterogeneous surface charge distribution can limit encapsulation efficiency, restrict tissue penetration, and complicate intracellular trafficking [170]. Furthermore, unlike nucleic acids such as mRNA, delivered proteins do not benefit from intracellular amplification, placing greater demands on cytosolic delivery efficiency and dose optimization [170–173].

Across dominant carrier platforms, including micelles, liposomes, emulsions, hydrogels, lipid nanoparticles (LNPs), solid lipid nanoparticles/nanostructured lipid carriers (SLNs/NLCs), and polymeric nanoparticles (Fig. 4), recurring translational bottlenecks are observed [42–51,160–170]. These include low loading efficiency for globular proteins, burst release or premature leakage, incomplete endosomal escape, complement activation or innate immune stimulation, and scale-up reproducibility challenges [170]. Importantly, the clinical validation of nanocarriers in oncology and mRNA vaccines has de-risked certain regulatory and manufacturing pathways; however, successful protein delivery generally requires cargo-specific formulation strategies rather than direct platform transposition [160–168]. The sections that follow critically examine each major carrier class, emphasizing not only their enabling features but also the mechanistic limitations and emerging mitigation strategies that will shape future translation.

### 4.2. Micelles

Polymeric or surfactant micelles have been explored for decades to solubilize hydrophobic peptides and small proteins, shield labile motifs from protease recognition and cleavage, and enhance residence at mucosal surfaces (Fig. 4A) [174,175]. A prominent showcase for an FDA-approved micellar peptide drug is cyclosporine A—formulated as an aqueous nanomicellar ophthalmic solution (0.09% Cequa^®^/OTX-101) for keratoconjunctivitis sicca [176,177]. The nanomicelles (NCELL^™^) organize cyclosporine into a hydrophobic core, improving ocular surface penetration. Clinically, this improvement demonstrated an increase in tear production relative to vehicle in Phase 3 trials, yielding an FDA-approval in 2018 [176–178]. Mechanistically, micellar encapsulation circumvents the very low aqueous solubility of cyclosporine and may reduce irritation compared with oil-in-water emulsions [179].

Despite this success, micelles face intrinsic thermodynamic limitations. Because they exist in equilibrium with unimers, dilution below the critical micelle concentration (CMC), as occurs following systemic administration, may trigger disassembly and premature drug release [180,181]. This dilution instability has limited broader systemic peptide and protein applications. Crosslinked micelles and block copolymers with very low CMC values have been developed to mitigate this risk, though crosslinking may compromise biodegradability and regulatory simplicity. Additionally, micelles are inherently better suited for hydrophobic or amphiphilic peptides; hydrophilic globular proteins do not readily partition into hydrophobic cores, resulting in low encapsulation efficiency without additional engineering. Strategies such as hydrophobic tagging or charge/chemistry modifications can improve loading but may alter folding or activity [182,183]. Furthermore, surfactant-mediated interfacial stress during assembly can induce aggregation or partial unfolding of sensitive proteins [169]. Thus, while micelles remain valuable for hydrophobic cyclic peptides such as cyclosporine, their broader translation to complex protein therapeutics remains constrained.

### 4.3. Liposomes

Classical liposomes are uni- or multilamellar phospholipid vesicles that protect cargo from enzymatic degradation, alter biodistribution, and enable depot or intracellular delivery (Fig. 4B) [184–186]. Their size, charge, cholesterol content, and PEGylation can be tuned to balance circulation time, cellular uptake, and adjuvant co-encapsulation for vaccines [187–189]. Liposomes have a strong FDA approval record for small molecules, including amphotericin B (AmBisome^®^), doxorubicin (Doxil^®^), and irinotecan (Onivyde^®^). While liposomal formulations of therapeutic proteins/peptides are not established among FDA-approved liposomal drug products, success has been established outside the U.S., exemplified by mifamurtide (MEPACT^®^), approved by the European Medicines Agency (EMA) for pediatric osteosarcoma [190].

The translational barriers for protein-loaded liposomes are multifactorial. Encapsulation efficiency for large hydrophilic proteins is often low, particularly when passive loading strategies are employed, and adsorption at lipid interfaces can promote aggregation or structural perturbation [184–186,191]. PEGylated liposomes, while extending circulation time, may trigger accelerated blood clearance (ABC phenomenon) upon repeat dosing through anti-PEG IgM responses [192]. Complement activation-related pseudoallergy (CARPA) has also been observed with certain liposomal formulations, necessitating careful lipid composition optimization [193]. Strategies such as fusogenic lipid incorporation and surface ligand modification have improved intracellular delivery in preclinical systems, but endosomal escape for large proteins remains a limiting step [191].

### 4.4. Emulsions

Emulsion delivery platforms consist of immiscible oil–water phases stabilized by surfactants (Fig. 4C). Microemulsions (<100 nm) and nanoemulsions (<1000 nm) enhance absorption by increasing surface area, with microemulsions offering thermodynamic stability and nanoemulsions kinetic stability [194,195]. Although primarily used for topical small molecules, emulsions have enabled peptide delivery, most notably cyclosporine formulated as the oral microemulsion Neoral^®^, which improved bioavailability and consistency [196,197]. In ophthalmology, cyclosporine 0.05% is delivered as an oil-in-water emulsion (RESTASIS^®^) to enhance ocular residence [198,199]. Emulsions also function as biologics-compatible vaccine adjuvants, exemplified by the MF59 oil-in-water emulsion in FLUAD^®^, which enhances immune responses in older adults [200,201].

However, emulsions pose stability concerns for proteins due to interfacial stress at oil–water boundaries that can promote unfolding or aggregation [169]. Surfactant selection is therefore critical, as surfactant–protein interactions can destabilize tertiary structure. Beyond interfacial unfolding, emulsions face route- and scale-dependent physical instabilities including creaming/sedimentation, coalescence, and Ostwald ripening, which can alter droplet size distribution and increase protein exposure to destabilizing interfaces [202]. Emerging approaches such as Pickering-stabilized emulsions, which use solid particle stabilizers rather than molecular surfactants, have shown promise for reducing interfacial protein stress and improving physical stability [203,204].

### 4.5. Hydrogels

Hydrogels are crosslinked polymer networks with high water content that provide a biomimetic environment for stabilized delivery of labile peptide and protein therapeutics (Fig. 4D) [42,205]. Release kinetics can be tuned by controlling mesh size, degradation rate, and responsiveness to stimuli such as pH, temperature, or enzymes [206,207]. Synthetic (polyethylene glycol, poly(organophosphazene)) and natural (alginate, hyaluronic acid, fibrin) hydrogels function as injectable depots, topical scaffolds, or implantable matrices that enable sustained, localized delivery while reducing burst release and proteolysis [42,205].

Hydrogels benefit from mild formulation conditions that can preserve protein structure; however, challenges include controlling initial burst release and heterogeneous degradation in vivo, and diffusion constraints for large proteins unless mesh size is tightly optimized [206,208]. Additionally, in vivo release behavior can vary with local biological conditions such as enzymatic activity, fluid turnover, and immune infiltration [209], and implantable hydrogels can trigger a foreign body response with fibrotic encapsulation that alters effective diffusion and drug elution kinetics over time [210]. Clinically, hydrogel-based products are widely used for localized applications. FDA-approved examples include becaplermin (Regranex^®^) for diabetic ulcers [211,212], Tisseel^®^ and Floseal^®^ for hemostasis and protein delivery [211–214], and hyaluronic-acid hydrogels (Gel-Syn^®^) for joint therapy [215], highlighting translational maturity for localized delivery and tissue interface applications.

### 4.6. Lipid nanoparticles

Lipid nanoparticles (LNPs) are lipid-based carriers initially developed for nucleic acid delivery but are now increasingly explored for peptides and proteins. Their clinical success in mRNA vaccines such as Comirnaty^®^ and Spikevax^®^ established a scalable, regulatory-validated platform for biologic delivery [43–45,47,216]. LNPs typically comprise four components: an ionizable lipid, a helper phospholipid such as distearoylphosphatidylcholine (DSPC), cholesterol for structural stability, and a PEGylated lipid for colloidal control (Fig. 4E) [160–168]. Ionizable lipids become protonated within the acidic endosome, promoting membrane disruption and cytosolic release through lipid phase behavior and related mechanisms [171,172].

While nucleic acid encapsulation relies on efficient electrostatic complexation between polyanionic RNA and ionizable lipids, protein delivery presents distinct challenges. Nucleic acids are linear polyanions with high charge density that condense readily during mixing, whereas proteins are folded macromolecules with heterogeneous surface charge and defined tertiary structures, often yielding lower encapsulation efficiency without additional formulation engineering [170]. In addition, whereas mRNA is routinely formulated under ethanol-containing and acidic mixing conditions, proteins may be more sensitive to organic–aqueous interfaces and pH excursions, with increased risk of partial unfolding or aggregation [160–168]. Another fundamental distinction is intracellular amplification: A single mRNA molecule can generate many protein copies through translation, meaning modest cytosolic delivery efficiency may suffice for therapeutic effect. Delivered proteins, by contrast, do not self-amplify, increasing the required dose and lipid exposure. Consistent with this, endosomal escape remains a key limiting step for cytosolic protein delivery, and strategies to increase fusogenicity can trade off against tolerability [172,173]. Inflammatory/toxicity liabilities, including innate immune stimulation and complement-related reactions, have been highlighted as design constraints for certain lipid compositions and repeat dosing regimens [217–219]. Advanced formulations employ mixed cationic and ionizable lipids, charge-reversible or pH-sensitive lipids, and optimized mixing conditions to enhance protein association and endosomal escape while minimizing toxicity [220,221]. Encapsulation efficiency and stability remain persistent bottlenecks for globular proteins, frequently requiring cargo-specific lipid selection and process optimization (charge engineering or excipients). Shear, solvent exchange, freezing, and lyophilization can induce aggregation unless stabilizing excipients are incorporated [222–224]. Although preclinical studies have demonstrated LNP delivery of protein cargos such as oral insulin, designed ankyrin repeat proteins (DARPins), and CRISPR–Cas9 ribonucleoprotein complexes [220,221,225–227], no protein-encapsulated LNP has yet achieved FDA approval for systemic protein therapy.

### 4.7. Solid lipid nanoparticles and nanostructured lipid carriers

Solid lipid nanoparticles (SLNs) are composed of solid lipid matrices that enhance stability, controlled release, and tolerability—enabling oral or mucosal delivery of labile peptides and proteins (Fig. 4F) [228]. Nanostructured lipid carriers (NLCs) improve upon SLNs by incorporating mixed solid–liquid lipids to reduce crystallinity-driven drug leakage and low encapsulation efficiency (Fig. 4G) [229]. Preclinical studies, particularly with insulin, have demonstrated improved resistance to gastrointestinal proteolysis and enhanced pharmacodynamic responses following oral administration relative to free peptide [228,230]. These effects are thought to arise from a combination of lipid-mediated protection, interaction with intestinal membranes, and lymphatic transport mechanisms that partially bypass first-pass metabolism.

However, translation has been limited primarily by challenges in lipid polymorphism, recrystallization during storage, burst release, and scale-up reproducibility [231–233]. Solid lipids can undergo polymorphic transitions from metastable α- and β'-forms to more stable β-crystalline structures, which decreases lattice imperfections and can expel encapsulated molecules over time [234]. While NLCs mitigate this risk by incorporating liquid lipids that disrupt crystal packing, drug expulsion and burst release can still occur during long-term storage or temperature cycling [234]. Additionally, the hydrophobic lipid matrix inherently favors lipophilic cargos, making efficient encapsulation of hydrophilic proteins difficult without additional strategies such as ion pairing, adsorption-based loading, or hydrophobic modification of the protein—approaches that may increase loading efficiency but may also introduce instability during dilution or exposure to biological fluids [235–237].

Manufacturing considerations represent an additional barrier to clinical translation. Many SLN production methods, including hot high-pressure homogenization, expose biomacromolecules to elevated temperatures and shear forces that can compromise protein structure [231–233]. Cold homogenization approaches reduce thermal stress but often yield broader particle size distributions and lower encapsulation efficiencies, complicating reproducibility and scale-up [234]. From a regulatory perspective, batch-to-batch variability in lipid crystallinity, surfactant distribution, and particle morphology can significantly influence release kinetics and stability, necessitating stringent control of critical process parameters during GMP manufacturing.

Other research efforts have emphasized translatability through optimized lipid design and scalable manufacturing. Novel excipients such as polyglycerol fatty acid esters (PGFAs) and glyceryl behenate enhance structural flexibility [231–233], while continuous-flow microfluidization enables reproducible good manufacturing practice (GMP)-scale production [238,239]. Functionalization by using PEGylated surfactants, cell-penetrating peptides, and ligand lipids has demonstrated improved mucosal adhesion and targeted uptake [240–243], while pH-sensitive or enzyme-cleavable lipids have shown improvements in controlled release in the gastrointestinal tract or tumor sites. Additionally, hybrid lipid–polymer nanoparticles (LPHNs) now integrate polymer stability with lipid permeability, improving peptide loading and sustained delivery [241].

### 4.8. Polymeric nanoparticles

Polymeric nanoparticles (NPs), particularly poly(lactic-*co*-glycolic acid) (PLGA)-based systems, are widely studied for encapsulating labile peptides and proteins, enabling controlled or stimuli-responsive release while reducing burst effects through excipients and advanced fabrication methods (Fig. 4H) [48–51]. However, the double-emulsion solvent evaporation methods commonly used for hydrophilic protein loading expose cargo to water–organic interfaces that can induce aggregation and reduce recovery [169]. In addition, PLGA degradation can generate acidic microenvironments that destabilize pH-sensitive proteins, motivating the use of buffering salts, protective excipients, and alternative fabrication strategies [244].

Protein instability in PLGA systems can arise from several mechanisms including acidic microclimate formation, interfacial denaturation during emulsification, and covalent acylation of peptides by reactive polymer degradation intermediates [245,246]. Strategies to mitigate these effects include buffering salts, polymer chemistry optimization, and improved manufacturing methods. For example, microfluidic fabrication platforms can generate monodisperse PLGA nanoparticles and reduce shear-induced instability during protein encapsulation [246]. A notable example illustrating the manufacturing and commercialization challenges of polymer-based protein depots is Nutropin Depot^®^, a PLGA microsphere formulation of recombinant human growth hormone that was discontinued due to the significant resources required to continue manufacturing and commercialization despite clinical efficacy [247].

Polymeric nanoparticle platforms have nevertheless achieved strong clinical validation in vaccines using self-assembled protein nanoparticle architectures, including HPV virus-like particle vaccines GARDASIL^®^ 9 and recombinant spike protein nanoparticle vaccine Nuvaxovid^®^ (NVX-CoV2373) [248,249]. Beyond vaccines, polymeric nanoparticles continue to be explored for enzymes and hormones, though manufacturing reproducibility and nano–bio interface characterization remain key development and regulatory challenges [48–51].

### 4.9. Comparative analysis of drug carrier platforms

Drug carriers differ fundamentally in cargo protection, release control, scalability, and regulatory maturity (Table 5 summarizes the analysis of carriers described in Section 6). Micelles and emulsions primarily improve solubility of hydrophobic peptides but provide limited structural protection. Liposomes and LNPs offer encapsulation and protection from enzymatic degradation but face stability and leakage challenges. Hydrogels function as depot systems, enabling sustained local release rather than systemic circulation. Polymeric nanoparticles and SLNs/NLCs provide tunable release kinetics and mucosal delivery potential but encounter scale-up and reproducibility barriers. LNPs have achieved the greatest regulatory validation in biologics due to mRNA vaccine success, suggesting strong translational potential for protein therapeutics.

## 5. Routes of administration

### 5.1. Parenteral delivery

#### 5.1.1. Overview

**Fig. 5.**
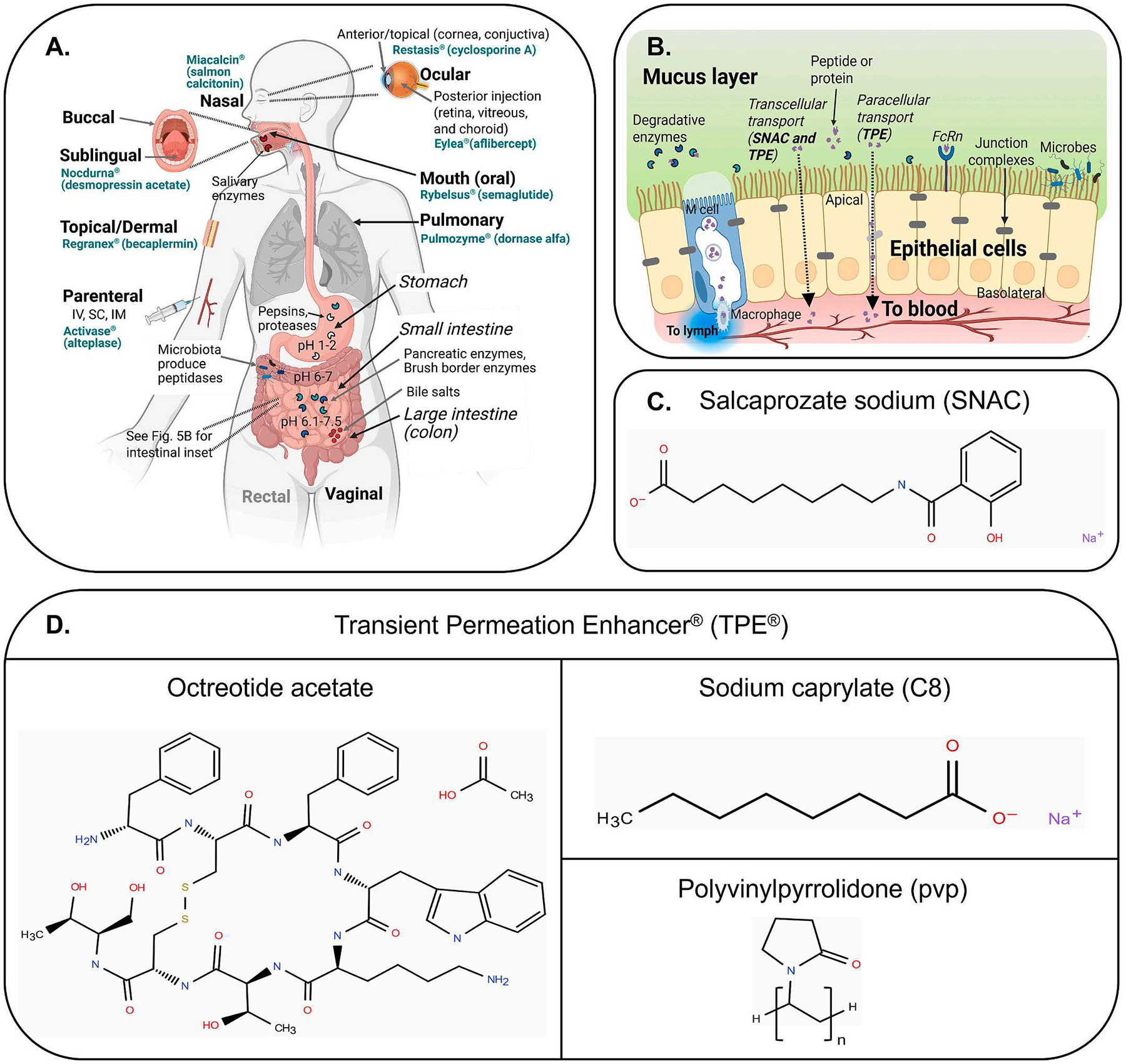
Oral protein/peptide delivery routes and transit in humans. (A) Major routes of administration shown, and key degradative organs in the GI tract (stomach, small intestine, large intestine). Representative drug example delivered by a particular route is shown in aqua text, if available. (B) Detailed transport across the mucus layer in the small intestine, where a peptide or protein may undergo transcellular or paracellular transport to traverse the epithelial layer or can be transported via transcytosis (mediated by M cells). (C) Structure for salcaprozate sodium (SNAC). (D) Structures for octreotide acetate, sodium caprylate (C8), and polyvinylpyrrolidone (pvp), the primary components of Transient Permeation Enhancer^®^ technology. Created in BioRender. LIM, C. (2026) https://BioRender.com/mc8vqck.

Parenteral administration, or delivery by injection, is the predominant administration route for protein and peptide therapeutics [250,251]. This approach bypasses oral delivery barriers such as enzymatic degradation, pH instability, and intestinal epithelial transport (Fig. 5) [16,250–252]. The most common parenteral routes are intravenous (IV), subcutaneous (SC), and intramuscular (IM) (Fig. 5A), while specialized approaches such as intrathecal and intra-articular delivery are used in select clinical contexts [253,254]. Select therapeutics are described in Table 6.

#### 5.1.2. Intravenous administration

IV administration provides immediate systemic exposure and complete bioavailability while avoiding first-pass metabolism [253]. These advantages make it indispensable in acute clinical settings but also introduce limitations, including the need for trained personnel, systemic adverse effects, and poor patient adherence for chronic therapy. IV delivery is critical in emergencies such as ischemic stroke, pulmonary embolism, and myocardial infarction. In these settings, recombinant tissue plasminogen activator alteplase is administered for rapid fibrinolysis [256–258]. Alteplase is a 527-amino-acid glycoprotein that converts plasminogen to plasmin but has a short plasma half-life of ~5 min [255,258]. To address this limitation, tenectaplase was engineered with point mutations that increased fibrin specificity and introduced altered glycosylation, reducing renal clearance and extending half-life approximately eightfold [274].

Short circulation half-life is common among IV-administered peptides and proteins, particularly those under ~40 kDa, which are rapidly cleared by renal filtration [275,276]. Chemical strategies such as PEGylation, glycosylation, and lipidation are widely used to increase hydrodynamic size and reduce clearance. Proteolytic degradation by circulating serine proteases, metalloproteases, and aminopeptidases further limits exposure [3]. DPP-IV, for example, rapidly degrades incretin hormones such as GLP-1 and GIP [25–27]. Clinically successful GLP-1 analogs, including liraglutide, semaglutide, and tirzepatide, combine fatty-acid lipidation to promote albumin binding with amino-acid substitutions that protect against DPP-IV cleavage, markedly extending half-life (Table 6) [28–32].

#### 5.1.3. Subcutaneous administration

Proteolytic degradation also affects SC and IM administration, where enzymatic activity in interstitial tissues can lead to non-linear pharmacokinetics and reduced systemic exposure [277,278]. SC delivery involves injection into adipose tissue, typically in the abdomen, thigh, or upper arm. Compared with IV administration, SC dosing allows self-administration, improves patient compliance, and reduces systemic adverse effects, although injection-site reactions are more common [279]. Insulin, first administered subcutaneously in 1922, represents the earliest clinical example of SC peptide therapy [280].

Recently, several biologics originally administered intravenously have transitioned to SC formulations, improving convenience and tolerability [281]. Examples include monoclonal antibodies such as trastuzumab, daratumumab, and rituximab, as well as immunoglobulin therapies (Table 6) [264,265,278,282–285]. This transition has been enabled by co-formulation with hyaluronidase, which degrades hyaluronan in the extracellular matrix, reducing tissue resistance and allowing larger injection volumes [286,287]. Historically, animal-derived hyaluronidases (bovine or ovine) were associated with immunogenicity and rare anaphylaxis due to impurities [288]. The FDA approval of recombinant human hyaluronidase (rHuPH20; ENHANZE^®^) in 2005 significantly reduced these risks, and its use in SC trastuzumab was approved in 2019 [41]. rHuPH20 has enabled safer, higher-volume SC dosing and is now widely used in protein and peptide formulations [41,289,290].

#### 5.1.4. Intramuscular administration

Intramuscular administration delivers drugs into large muscle groups such as the deltoid or gluteus, with absorption influenced by local blood flow [253]. A notable IM-administered biologic is clesrovimab, a recombinant IgG1 monoclonal antibody engineered with Fc mutations (M252Y/S254T/T256E) to enhance neonatal Fc receptor binding and extend half-life [268]. Clesrovimab targets the respiratory syncytial virus (RSV) fusion protein and was FDA-approved in 2025 for RSV prevention in infants entering their first RSV season [267].

#### 5.1.5. Other routes of parenteral administration

Specialized parenteral routes are used when systemic delivery cannot achieve sufficient tissue exposure. Intrathecal administration enables direct delivery to the central nervous system, bypassing the blood-brain barrier. Ziconotide, a 25-amino-acid conopeptide derived from *Conus magus*, is administered intrathecally for severe pain management because it does not cross the blood-brain barrier (BBB) when given intravenously [254,270–273]. Intrathecal trastuzumab has also been explored for HER2-positive breast cancer with brain metastases, with reports of neurological improvement and acceptable tolerability [291]. However, intrathecal delivery carries risks including infection and the need for indwelling devices. Intra-articular injection is another emerging parenteral route, particularly for osteoarthritis. Clinical studies have evaluated formulations such as Prostrolane^®^, containing hyaluronic acid and synthetic peptides, and BPC-157, a pentadecapeptide derived from gastric secretions [292,293]. These studies remain small, and no peptide-based intra-articular therapies are FDA-approved, highlighting the need for further investigation. This is likely due to lack of sufficient large scale human testing, regulatory issues, and non-standardized manufacturing issues, to name a few [294].

### 5.2. Oral delivery

#### 5.2.1. Overview

Oral delivery of any drug is considered the “holy grail” [295] for the pharmaceutical industry due to patient compliance, safety profile, and ease of manufacture. However, less than 2% of FDA-approved peptide and protein drugs are formulated orally (Fig. 2B), due to inefficient absorption and destruction during transit through the gastrointestinal (GI) tract [296], shown in Fig. 5A.

#### 5.2.2. Barriers to delivery through the GI tract

Peptide and protein transit and degradation in the GI tract are depicted in Fig. 5A. While peptide and protein drugs encounter salivary peptidases and digestive enzymes in the mouth (oral cavity) [297], drugs to be swallowed whole have minimal digestion in the mouth and esophagus [298]. However, in the stomach, acid proteases, mainly pepsin (and pepsin C and cathepsin D) can degrade proteins [299,300]. In the small intestine, enzymes secreted by the pancreas (trypsin, chymotrypsin, elastase, and carboxypeptidases) [301] and brush border peptidase enzymes (aminopeptidases, carboxypeptidases, endopeptidases, and dipeptidases) [302] are in responsible for breakdown of peptides and proteins. Bile salts in the small intestine disrupt protein structure, making proteins more susceptible to degradation [303]. Finally, proteolytic degradation in the large intestine is promoted by microbiota (predominantly Bacteriodes spp) that produce proteases including aminopeptidases, dipeptidyl peptidases, and carboxypeptidases [304,305].

The GI tract has a wide pH variability, ranging from 1 to 7.5 [306,307], acidic in the stomach (pH 1–2) and more basic (pH 6–7.5) in the small and large intestines (Fig. 5A) [307]. The changing pH through the GI tract poses problems for oral peptide and protein drugs due to their stabilities at different pHs (e.g., exenatide is most stable from pH 4.5–5.5) [306–308]. Many pH-dependent chemical degradation reactions and pH-dependent physical instabilities are well documented [309]. The pH stability ranges have been compiled for 300+ proteins, ranging from 4 to 8 for most [310]. This “marginal stability” is also impacted by concentration, ionic strength, temperature [311], amino acid composition and 3D structure [310,312].

The major site of drug absorption is from the small intestine with a surface area of 30m^2^ [313,314], the area of a large U.S. household bedroom. In the small intestine, the duodenum and jejunum are major sites of absorption of peptides/proteins [315]. The small intestine cellular epithelial layer is overlayed by a mucus barrier, a viscoelastic, adherent gel layer of negatively charged mucin glycoproteins, secreted primarily by goblet cells (Fig. 5B) [316]. The mucin layer (in the stomach and large intestine), acts as a physical barrier, slows diffusion of trapped molecules, and binds to proteins [317]. Mucin allows passage of nutrients but keeps large molecules such as pathogenic bacteria (or proteins) from entering [315]. Mucus-penetrating strategies such as PEGylated nanoparticles, mucoadhesive, and mucolytic approaches are well-reviewed elsewhere [318]. Once past the mucus, the main cell type in the small intestine is columnar enterocytes (Fig. 5B; yellow cells), while M cells (Fig. 5B; blue cell), with their own drug delivery potential, make up a much smaller fraction. Transport of drugs across the epithelial layer (enterocytes) can occur via transcellular (through cells) or paracellular (between cells through tight junctions) mechanisms (Fig. 5B) [317].

#### 5.2.3. Transport through the GI tract

Passive transcellular transport is limited to peptides <1kDA (~ 9 amino acids) and lipophilic peptides. Cyclosporine (cyclosporine A), an 11 amino acid cyclic fungal peptide, utilizes the transcellular passive pathway [306,319,320] (Table 7A). Cyclosporine was later reformulated as Neoral^®^ (Table 7A) to improve bioavailability and can be taken without regard to meals [59,321,322]. An exciting development for oral delivery of peptides is exemplified by Rybelsus^®^ (semaglutide) in Table 7A, a 31 amino acid GLP-1 receptor agonist for type 2 diabetes [321,322]. Semaglutide, while structurally similar to GLP-1, has been chemically modified to extend its half-life via lipidation and PEGylation of the molecule. An oral alternative to injectable insulin or other GLP-1 agonists is of great interest due to the large market potential (improved patient compliance vs. injectable), not only for diabetes, but as weight loss agents. Oral Rybelsus^®^ is formulated with absorption enhancer SNAC to improve passive transcellular transport by improving potency, stability, lipophilicity, and half-life (Fig. 5C) [34]; it also lowers gastric pH to reduce cleavage by pepsin and improves absorption in gastric epithelial cells (rather than the intestines) [35]; Table 7A. Oral semaglutide is in OASIS-1 Phase 3 clinical trials for weight loss [320]. Other oral peptides in the pipeline are listed in Table 7D.

Active (or carrier-mediated) transcellular transport can accommodate larger peptides/proteins using receptor-mediated processes. For example, FcRn (Fig. 5B) binds the Fc domain of IgG as its natural ligand and can act as a transporter for larger protein or peptide substrates [33,36]. Notable protein therapeutics that exploit FcRn targeting and highlight the success of this pathway include Enbrel^®^ (etanercept), Orencia^®^ (abatacept), and Eylea^®^ (aflibercept) [114,116,323,324]. Oral delivery of peptides/proteins via milk-derived exosomes (small extracellular vesicles) show promise with absorption via FcRn [325]. GLP-1 agonist liraglutide and insulin have both been separately encapsulated in milk exosomes for oral drug delivery. Lastly, peptides/proteins can also exploit various receptor mediated transport mechanisms using the PEPT1 (oligopeptide) transporter (although not transcellular) [326], transferrin receptor [327], vitamin B12 receptor [328], and folate receptor-decorated nanoparticles [329].

The paracellular route transports molecules between epithelial cells which are held together by epithelial junction complexes (Fig. 5B), including tight junctions (near apical membrane), adherens junctions (middle), and desmosomes (near basolateral membrane) [330]. Tight junctions form <1 nm pores that only allow molecules under 600 Da (~5–6 amino acids) to pass through [331]. The 9 amino acid synthetic analog of vasopressin, desmopressin (oral tablet formulation; Table 7A), uses the paracellular route for absorption, but is still poorly absorbed with low bioavailability (0.5%) [34]. Orally delivered octreotide (Mycappsa^®^), a cyclic modified peptide analog of somatostatin (Table 7A), uses Transient Permeation Enhancer^®^ technology (Fig. 5D) [332] with proposed mechanisms including temporary loss of tight junction structural integrity to enhance paracellular delivery [332] and transcellular delivery with surfactant-like properties [333]. A much rarer cell type, M cells (antigen sampling microfold cells), comprise only 1% of small intestine cells (blue cell in Fig. 5B), found in specialized Peyer's patches in the intestinal wall. M cells have a thinner mucus layer and allow sampling of contents by the immune system (macrophage cells) that traffic to the lymph fluid [315], which can be exploited for drug delivery. M cell transcytosis can occur by receptor- and non-receptor mediated uptake [334]. Oral delivery via M cell pathways has been mostly restricted to vaccine delivery using attenuated or inactivated pathogens [335].

### 5.3. Topical and transdermal delivery

#### 5.3.1. Overview

**Fig. 6.**
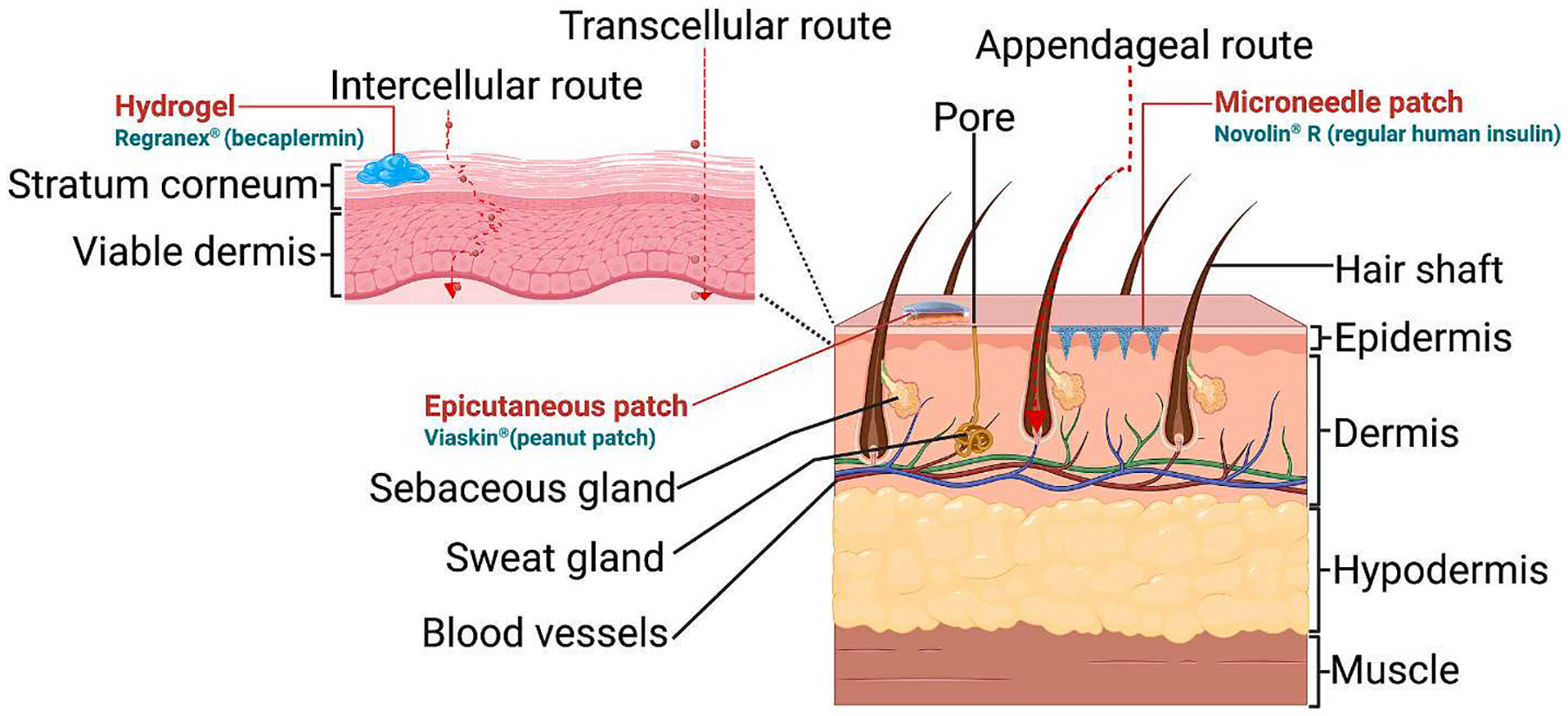
Anatomy of the human skin and the principal pathways of transdermal and topical transport. The left panel provides a magnified view of the stratum corneum and viable epidermis, illustrating transcellular and intercellular diffusion pathways. The main schematic (right) depicts the full anatomical organization of the skin, including the epidermis, dermis, hypodermis, and associated appendages and shows the appendageal route of permeation through hair follicles and glands. Representative drug example(s) delivered by a particular system are shown in aqua and red text respectively. Created in BioRender. LIM, C. (2026) https://BioRender.com/wfni7py.

The human skin is a multilayered organ that plays a crucial role in maintaining homeostasis and helps protect the body from the external environment [365]. It is the body’s largest organ and is composed of three main layers: the epidermis (composed of the stratum comeum and viable epidermis), dermis, and hypodermis (Fig. 6) [366,367]. Skin is a non-invasive and accessible route for both topical and transdermal delivery of protein and peptide therapeutics, offering advantages such as improved patient compliance, avoidance of first-pass metabolism, and the potential for sustained or localized release. Although often grouped together, topical and transdermal delivery represent distinct strategies with different therapeutic goals [368]. Topical delivery targets the skin itself, aiming to achieve a localized effect within the epidermis or dermis without significant systemic absorption. This is especially relevant for dermatologic conditions such as psoriasis, atopic dermatitis, or chronic wounds, where peptides and proteins modulate local inflammation, matrix remodeling, or immune signaling. For instance, becaplermin (Regranex^®^, Fig. 5A), a recombinant human protein growth factor (rh-PDGF-BB), is delivered via a gel directly to the wound bed of diabetic neuropathic ulcers, where it acts on keratinocytes and fibroblasts at the injury site, thereby bypassing the need for penetrating the skin (Table 8A) [369,370]. In contrast, transdermal delivery aims to enable systemic absorption, bypassing the skin barrier to reach the bloodstream [369].

#### 5.3.2. Barriers to macromolecular delivery

Although the barrier function of the skin is vital for protection, it significantly limits the delivery of macromolecules. The stratum corneum serves as the principal mechanical barrier to the permeation of drugs in transdermal drug delivery. It has been likened to a “brick and mortar” model and its structural rigidity and low water content results in a hydrophobic environment that severely limits the penetration of hydrophilic compounds such as peptides and proteins while allowing the passage of only small (<500 Da), lipophilic molecules [371–373]. Despite these constraints, several FDA-approved topical peptides and proteins can achieve therapeutic benefit through localized action within superficial skin layers. Recothrom^®^ (Table 8A) acts on the surface of the wound during surgical hemostasis [374,375], eliminating the need for systemic absorption, while Viaskin peanut epicutaneous immunotherapy patch (Table 8B), an investigational drug being developed for topical application, uses an occlusive environment to deliver lyophilized peanut protein to Langerhans cells within the viable epidermis [376]. Because these antigen-presenting cells reside above the deep barrier layers, Viaskin avoids deep stratum corneum penetration yet achieves antigen presentation and immune modulation. Beneath the stratum corneum, the viable epidermis presents another barrier to drug delivery, and it comprises several stratified layers: the stratum basal, stratum spinosum, and stratum granulosum [368,377]. Tight junctions in the granular layer of the epidermis form a secondary barrier that regulates paracellular transport by sealing adjacent cells and limiting the diffusion of molecules based on size and charge. While certain ligands can interact with proteins (claudins, occludins) in the tight junctions to modulate permeability, the ability to transiently enhance permeability without compromising barrier integrity remains a key challenge [378–381].

The basement membrane acts as an additional barrier to transdermal drug delivery. Its sheet-like composition of laminins, collagens and proteoglycans acts as a molecular sieve that slows the diffusion of larger therapeutic agents [366,382,383]. The dermis is a connective tissue-rich layer underlying the epidermis. It is composed of a dense extracellular matrix of collagen and elastin fibers and houses a dense network of blood and lymphatic vessels, nerves, sweat glands, sebaceous glands, and hair follicles [365,384]. The capillary network within the dermis represents the final barrier before drugs can enter systemic circulation. Endothelial cells lining these vessels control molecular transport between the bloodstream and surrounding tissue. Their permeability is responsive to physiological conditions, including variations in pressure and temperature, which can influence blood flow and drug absorption rates. Enhanced perfusion facilitates systemic uptake, while reduced flow may limit bioavailability [380,385,386]. Since most peptides and proteins cannot passively permeate the hydrophobic, tightly packed lipid matrix of the stratum corneum due to the various barriers the skin poses to the delivery of these drugs, physical penetration enhancement technologies are needed to attain systemic delivery. Microneedles represent one of the most impactful innovations in this space as they create micron-scale aqueous channels across the stratum corneum, bypassing intercellular lipids and tight junctions. Several investigational products exemplify this strategy. For instance, human regular insulin (Novolin^®^ R, Table 8C) uses short solid microneedles and an iontophoresis patch to deliver bolus insulin on demand [387]. Another transdermal microneedle patch technology uses dissolving CaCOa/Polyvinylpyrrolidone (PVP) microneedle matrices (Table 8C) to deliver insulin [388].

#### 5.3.3. Transport of compounds through the skin

Compounds applied to the skin permeate primarily through one of three pathways (left panel, Fig. 6). The intercellular lipid pathway is the major route for molecular diffusion, and it involves diffusion through the continuous lipid bilayers between corneocytes, which poses a significant barrier to hydrophilic molecules such as peptide and proteins [389]. The transcellular (intracellular) route involves drug transport through the corneocytes themselves, which requires alternating diffusion through hydrophilic (cellular/corneocytes) and lipophilic (membrane) environments [390]. The allergen in the Viaskin peanut patch (Table 8B) [376] primarily uses the transcellular route to permeate through the superficial layers of the intact epidermis by passing through the cells of the stratum corneum into the viable epidermis where they are captured by Langerhans cells [391,392]. The appendageal pathway allows drug delivery through structures such as hair follicles and sweat glands which enables drugs to bypass the dense structure of the stratum corneum, allowing for localized delivery to deeper skin layers [393,394]. Table 8 provides an overview of peptides and proteins that have received FDA approval for topical use, along with a selection of candidates currently under investigation for both topical and transdermal applications.

### 5.4. Intranasal delivery

Intranasal delivery (Fig. 5A) is a non-invasive, patient-friendly route that enables rapid systemic absorption due to the high vascularization and thin epithelial barrier of the nasal mucosa. In addition to systemic delivery, intranasal administration can enable direct nose-to-brain (NTB) transport via olfactory and trigeminal pathways, bypassing the BBB [401–403]. Despite these advantages, the nasal cavity presents substantial limitations for peptide and protein therapeutics. The absorptive surface area is limited (~150 cm^2^), mucociliary clearance rapidly removes deposited formulations, and enzymatic proteolysis combined with tight junctions severely restrict macromolecular permeability, often resulting in <1% bioavailability for unmodified peptides [401,402,404].

To address these and other barriers (similar barriers depicted in Fig. 5B), intranasal peptide formulations frequently rely on absorption enhancers, stabilizing excipients, or carrier systems (Table 9A). Noctiva^™^ incorporates cyclopentadecanolide (CPD), a permeation enhancer that transiently loosens epithelial tight junctions to facilitate desmopressin uptake [401,402,404]. Cyclodextrins are also used to improve aqueous solubility and protect peptides from enzymatic degradation within nasal mucus [405]. Similarly, Baqsimi^®^, the first FDA-approved intranasal dry-powder glucagon for severe hypoglycemia [406], employs β-cyclodextrin to stabilize glucagon at room temperature [407,408] and dodecylphosphocholine (DPC) to lower epithelial resistance and promote paracellular transport [409]. Beyond excipient-based approaches, mucoadhesive nanocarriers have been investigated to prolong nasal residence time, shield peptides from enzymatic degradation, and enhance epithelial transport via transient tight junction opening or endocytosis [410–412]. These strategies are particularly relevant for NTB applications, where localized delivery to the central nervous system is desirable. Clinical interest in NTB delivery is illustrated by a Phase 3 study evaluating intranasal carbetocin for Prader–Willi syndrome, which showed early improvements in hyperphagia-related behaviors [413]. Similar approaches are under investigation for neurological and neurodevelopmental disorders, including Alzheimer's disease, schizophrenia, autism spectrum disorder, and Down syndrome [401,414–416].

Future advances in intranasal delivery are likely to focus on dry-powder devices, mucoadhesive depots, and NTB-targeted carriers. Clinical success will depend on demonstrating clear advantages over injectable formulations, ensuring room-temperature stability, and maintaining acceptable safety profiles for chronic outpatient use.

### 5.5. Pulmonary delivery

Pulmonary delivery exploits the lung's exceptionally large surface area (~75 m^2^), ultra-thin alveolar epithelium (0.1–0.5 μm), and dense capillary network to enable rapid systemic absorption of peptides and proteins [452], avoiding gastrointestinal degradation and hepatic first-pass metabolism. However, inhaled biologics must overcome several anatomical and physiological barriers. Particles deposited in the conducting airways may be cleared by mucociliary transport before reaching the alveoli (Fig. 5A), while alveolar macrophages, enzymatic degradation, and surfactant binding can limit bioavailability [452,453]. The alveolar epithelium itself remains a permeability barrier for macromolecules [452].

Formulation strategies to address these challenges include encapsulation within micro- or nanoscale carriers, co-formulation with absorption enhancers or protease inhibitors, and stabilization against shear and aerosolization stresses [452,454] (Table 9B). Exubera^®^, the first FDA-approved inhaled insulin, demonstrated clinical efficacy but failed commercially due to device bulk, cost, and limited adoption [455]. Afrezza^®^ improved upon this concept through a combination of formulation and device innovation, using technosphere^®^ microparticles composed of fumaryl diketopiperazine (FDKP), capable of self-assembly into 2–2.5 μm particles that protect insulin during delivery and rapidly dissolve in the alveolar environment [423,425]. This results in ultrafast insulin absorption, with peak plasma levels achieved within 12–15 min—substantially faster than subcutaneous insulin [425,456].

The compact, disposable “Dreamboat” inhaler further improved usability and patient acceptance, contributing to better adherence in clinical studies [457]. Together, rapid pharmacokinetics and user-friendly design position Afrezza^®^ as a viable alternative for mealtime glucose control. Building on this success, next-generation pulmonary biologics are being developed with advanced nanocarriers and inhaler platforms designed for precise lung deposition, controlled release, and improved stability [458]. Continued progress in device engineering and formulation science is expected to expand the scope of inhaled peptide and protein therapeutics.

### 5.6. Sublingual and buccal delivery

Sublingual (SL) delivery involves absorption across the thin, highly vascularized mucosa beneath the tongue, or via the inner cheek membrane for buccal delivery (Fig. 5A), providing rapid systemic exposure while bypassing gastrointestinal degradation and first-pass metabolism [403]. This route improves patient compliance, particularly for individuals who wish to avoid injections or have difficulty swallowing. However, SL delivery is constrained by limited surface area, continuous saliva flow that dilutes formulations, and enzymatic activity within saliva and mucosal tissues that degrade peptides and proteins [403]. Clinically, SL delivery has been successfully applied to low-dose peptides and protein-based products (Table 9C). NOCDURNA^®^ is a rapidly dissolving SL lyophilisate that delivers microgram quantities of a peptide analog with efficient absorption [403]. Ragwitek^®^, an orally disintegrating SL tablet containing ragweed pollen extract, enables daily allergen immunotherapy without injections, demonstrating that even large protein allergens can be administered sublingually [459].

To overcome residence-time limitations, mucoadhesive and structured delivery systems are under active investigation. Chitosan-hyaluronic acid patches have been shown to adhere beneath the tongue, extend contact time, and improve protein penetration while preserving bioactivity [460]. Electrospun nanofiber films with saliva-repelling backing layers reduce washout and direct peptide transport into mucosal tissue [461]. A bioinspired “octopus sucker” patch that mechanically anchors to the buccal (cheek) mucosa while incorporating permeation enhancers increased desmopressin bioavailability by approximately 100-fold compared to conventional tablets, with favorable tolerability demonstrated in a first-in-human study [462]. These technologies address the principal limitations of SL and buccal delivery and may broaden its applicability to a wider range of peptide and protein therapeutics.

### 5.7. Ocular delivery

#### 5.7.1. Overview

Topical eye drops remain the primary modality for anterior segment diseases due to ease of access, patient tolerability, and minimal systemic exposure [463]. However, tear turnover, blinking, and nasolacrimal drainage rapidly remove most of an applied dose, while the corneal epithelium imposes a tight paracellular barrier (~2 nm pore size) [463]. The conjunctiva is more permeable but favors systemic absorption due to its vascularization [463,464]. Proteolytic enzymes in tears and metabolic activity in anterior tissues further degrade peptides/proteins, making therapeutic levels hard to sustain without frequent dosing or specialized formulations [463–465].

Ocular delivery can target disease either the anterior (cornea and conjunctiva) or posterior segments (retina, vitreous and choroid), depicted in Fig. 5A each of which presents distinct biological barriers [430,433,463–465]. Each region presents its own biological barriers, from tear turnover and blinking at the front of the eye to tightly regulated blood-retina-barrier (BRB) at the back. Drug design must overcome these challenges while achieving therapeutic levels.

#### 5.7.2. Anterior segment delivery (cornea and conjunctiva)

Topical eye drops remain the primary modality for anterior segment diseases due to ease of access, patient tolerability, and minimal systemic exposure [463]. However, tear turnover, blinking, and nasolacrimal drainage rapidly remove most of an applied dose, while the corneal epithelium imposes a tight paracellular barrier (~2 nm pore size) [463]. The conjunctiva is more permeable but favors systemic absorption due to its vascularization [463,464]. Proteolytic enzymes in tears and metabolic activity in anterior tissues further degrade peptides/proteins, making therapeutic levels hard to sustain without frequent dosing or specialized formulations [463–465].

Strategies to improve anterior segment delivery focus on increasing residence time and permeability. Bioadhesive polymers, viscosity enhancers, and cationic emulsions slow tear washout and enhance corneal contact, while penetration enhancers and cell-penetrating peptides transiently loosen tight junctions [463–465]. Clinical examples include cyclosporine A emulsion for dry eye disease (Fig. 5A) and cenegermin (recombinant human nerve growth factor) eye drops for neurotrophic keratitis (Table 9D) [430,433]. These products demonstrate that, with appropriate formulation, topical delivery of peptides and proteins to the anterior eye is clinically feasible.

#### 5.7.3. Posterior segment delivery (retina, vitreous, and choroid)

Delivery to the posterior eye is significantly more challenging due to the blood–retinal barrier (BRB), which severely restricts systemic entry of hydrophilic macromolecules [465]. Topical delivery is similarly ineffective, with only a minute fraction of a dose reaching the vitreous or retina [464]. Consequently, intravitreal injection remains the gold standard for delivering peptides, proteins, and antibodies to posterior structures. Intravitreal administration achieves high local drug concentrations while minimizing systemic exposure, and all FDA-approved biologics for retinal disease use this route [464]. (Table 9E). However, it is invasive, requires repeated procedures due to limited intraocular half-lives (hours to days), and carries risks such as infection, hemorrhage, and retinal detachment [464,465].

Implantable systems such as the Port Delivery System for ranibizumab provide continuous drug release over months, achieving outcomes comparable to monthly injections with far fewer procedures [444]. Protein engineering approaches, such as the VEGF-trap fusion protein aflibercept (Eylea^®^, Fig. 5A), leverage high-affinity binding and IgG-like stability to extend intraocular half-life and potency [323,324]. Emerging technologies include magnetically guided ocular microrobots that can be injected and navigated within the eye to deliver drugs directly to disease sites [466–468]. Early studies demonstrate rapid clearance of vitreous hemorrhage and precise localization, with first-in-human feasibility trials planned [464]. Although still experimental, such approaches highlight the potential for highly targeted, minimally invasive ocular drug delivery.

### 5.8. Vaginal delivery

The vaginal route offers a highly vascularized mucosa, avoidance of first-pass metabolism, and the ability to achieve high local drug concentrations with reduced systemic exposure [469,470]. However, acidic pH, variable microbiota, proteolytic activity, and mucus turnover complicate peptide and protein delivery, with performance influenced by hormonal cycles and patient-related factors [469,470]. Formulation strategies emphasize residence-time extension, protection from degradation, and controlled release. An example is an interferon-α2b vaginal suppository for high-risk HPV lesions, which demonstrated localized efficacy with limited systemic exposure [471]. Its gel formulation embeds the protein in a protective base that melts in situ, localizing drug near the cervix, limiting systemic exposure, and improving viral clearance in clinical studies [471]. These results highlight that with protective formulations, vaginal delivery of peptide or protein therapeutics is feasible. Market interest is rising with persistent unmet needs in HPV-related disease, bacterial vaginosis, candidiasis, and atrophy. However, adoption will hinge on safety (irritation, microbiome preservation) [472], user acceptability (discreet, comfortable, low-mess formats) [473,474], dose uniformity, and scalable manufacturing. Near-term innovation will likely prioritize long-acting rings and films, muco-inert or muco-penetrating nanocarriers, and smart gels that balance adhesion with biocompatibility [469,470].

### 5.9. Rectal delivery

Rectal delivery partially bypasses hepatic first-pass metabolism but suffers from low patient acceptance and highly variable absorption [403], Clinical studies with rectal insulin showed modest bioavailability (~25% of injectable dosing) and required high doses, leading to abandonment of this route for peptide therapeutics [475,476]. Consequently, rectal delivery remains unlikely to support widespread clinical use of peptide or protein drugs.

### 5.10. Comparative analysis of delivery routes

Routes of administration reflect a hierarchy of biological barriers that determine systemic exposure, onset of action, and translational feasibility for peptide and protein therapeutics (Table 10). Parenteral routes remain the dominant approach because they bypass proteolysis and epithelial transport barriers that severely limit macromolecular absorption. Intravenous delivery provides immediate systemic exposure and complete bioavailability, making it well suited for antibodies, enzyme replacement therapies, and oncology biologics. Subcutaneous injection represents the most widely adopted compromise between pharmacokinetic reliability and patient convenience, enabling self-administration and sustained systemic exposure through lymphatic uptake, particularly when combined with half-life extension strategies or co-administration with hyaluronidase. Intramuscular delivery is commonly used for vaccines and depot formulations, while specialized routes such as intrathecal, intravitreal, or intra-articular administration enable localized delivery to protected anatomical compartments including the central nervous system, retina, or synovial space.

Non-parenteral routes are actively pursued to improve patient adherence but face substantial physiological barriers. Oral delivery remains the most desirable route because of its unmatched convenience and patient acceptance, yet it presents the most complex barrier environment for macromolecules. After ingestion, peptide and protein drugs encounter sequential obstacles including acidic gastric pH, enzymatic degradation by proteases throughout the gastrointestinal tract, diffusion limitations within the mucus layer, restricted paracellular transport across tight junctions, and first-pass hepatic metabolism. Together, these factors result in extremely low intrinsic bioavailability for most biologics. Consequently, current oral delivery strategies rely on mechanistically distinct technological solutions. Chemical approaches employ permeation enhancers, enzyme inhibitors, and formulation excipients that transiently modify epithelial permeability or protect cargo from degradation. Structural formulation strategies, including lipid-based carriers, nanoparticles, and mucoadhesive systems, aim to shield proteins during transit and promote epithelial uptake. In contrast, emerging device-based technologies such as ingestible microneedle or self-orienting injection capsules bypass epithelial transport barriers entirely by mechanically delivering the drug across the intestinal wall. Although these approaches have enabled the first clinically approved oral peptide formulations, systemic exposure remains highly formulation dependent and generally lower than that achieved through parenteral administration.

Topical and transdermal delivery are effective for localized dermatologic therapy but rarely achieve systemic exposure without physical enhancement technologies such as microneedles or electroporation. Intranasal delivery offers rapid absorption and potential access to the central nervous system through olfactory pathways but is limited by mucociliary clearance and restricted dosing volumes. Pulmonary delivery provides a large absorptive surface and thin epithelial barrier that can enable systemic uptake of certain peptides, although device complexity and variability in inhalation performance remain challenges.

Additional mucosal routes provide more specialized opportunities. Sublingual and buccal delivery bypass gastrointestinal degradation and hepatic first-pass metabolism but are limited by small absorptive surface area and salivary dilution. Ocular delivery is primarily used for localized treatment of retinal disease, typically requiring intravitreal injection to overcome corneal and blood–retinal barriers. Vaginal and rectal delivery can support localized therapy or limited systemic absorption through mucosal tissues but remain constrained by variability in permeability and patient acceptance. Collectively, route selection reflects a balance between barrier complexity, therapeutic objective (local versus systemic exposure), pharmacokinetic requirements, and patient convenience.

## 6. Insight and outlook

### 6.1. The status of peptide and protein drug delivery in 2026

#### 6.1.1. Overview

Peptide and protein therapeutics are entering a matured, platform-oriented era in which the trajectory for clinical success is no longer dictated by target interactions alone, but also by (i) molecular engineering to enhance intrinsic stability and half-life, (ii) formulation science to enable high-concentration and extended-action, and (iii) device-enabled self-administration to improve patient compliance and shift care from the clinic to the home. Over the past decade, the field has begun to incorporate system-level design into earlier development, emphasizing route of administration, formulation microenvironment, practical delivery devices, and manufacturability alongside classical liabilities like proteolysis, clearance, and immunogenicity [7]. This philosophical design shift is exemplified by the move toward more patient-centric dosing of weekly/monthly regimens using fixed-dose pens and wearables, and noninvasive administration routes, reflecting lessons learned from both monoclonal antibody and metabolic peptide markets.

#### 6.1.2. Monoclonal antibodies and entero-pancreatic hormone-based peptides

Monoclonal antibodies and entero-pancreatic hormone–based peptides illustrate how delivery considerations are now steering the trajectory of the entire field. For monoclonal antibodies, the primary delivery challenge is no longer systemic bioavailability, which is essentially complete following parenteral administration, but rather dose, volume, frequency, and patient experience. The historical reliance on intravenous infusion is giving way to high-concentration subcutaneous formulations that enable home administration and reduce healthcare burden. This transition has forced major advances in formulation science, particularly around viscosity control, protein–-protein interactions, and colloidal stability at concentrations often exceeding 100–200 mg/mL [264,265,278–285]. As a result, delivery feasibility is now tightly coupled to antibody sequence engineering, isoelectric point optimization, and excipient selection, demonstrating that delivery constraints increasingly propagate upstream into molecular design [48–51,264,265,281–287].

The emergence of large-volume subcutaneous delivery further exemplifies this shift. Many next-generation antibodies and antibody-derived formats, such as bispecifics, require doses that exceed the comfortable limits of traditional SC injections. This has catalyzed the adoption of on-body injectors and wearable delivery devices, which decouple dose volume from injection pain and duration [41,266,286–290]. Importantly, these devices are no longer peripheral accessories but co-development partners that influence formulation viscosity limits, fill–finish strategies, and regulatory comparability plans.

In parallel, entero-pancreatic hormone–based peptides, particularly GLP-1 and dual GLP-1/GIP receptor agonists such as semaglutide and tirzepatide, have redefined what is commercially and clinically possible for peptide therapeutics. These molecules demonstrate how chemical modification strategies like lipidation, Fc and albumin fusion, and backbone stabilization can be combined to achieve once-weekly or longer dosing while preserving potent pharmacology [56–58,277]. The success of these therapies has normalized chronic peptide administration and has strengthened the economic and translational case for investing in delivery-enabled peptide optimization across multiple disease areas [15–17,21–23].

The innovations critical to these peptides’ success extend beyond half-life extension alone. For example, oral semaglutide represents a rare but important proof-of-principle for oral peptide delivery, achieved through a formulation-centric strategy using absorption enhancers rather than a universal nanoparticle delivery platform [33,36]. While this success should not be overgeneralized and oral delivery will likely remain limited to potent, low-dose peptides, it has reshaped expectations by showing that localized, mechanism-specific solutions can succeed where universal platforms have struggled [34,36,59,295,296,320–322,477].

#### 6.1.3. Oral delivery

Oral delivery approaches are consolidating around a limited set of clinically realistic strategies: (i) permeation enhancers that act transiently and locally, (ii) protective formulation microenvironments that stabilize macromolecules in harsh GI conditions, and (iii) device-assisted approaches that bypass epithelial transport constraints altogether. Recent reviews of permeation enhancers emphasize a shift away from single-agent solutions toward new chemistries and combinations that balance efficacy with epithelial recovery and mucosal safety [345,478]. At the mechanistic level, recent research has increasingly focused on quantitatively understanding how absorption enhancers interact with epithelial membranes and how peptides partition into these temporarily permeabilized regions. This work reinforces the view that oral bioavailability arises from the combined influence of formulation chemistry, peptide physicochemical properties, and epithelial membrane biophysics [306–311,313–320]. Current research is therefore shifting toward next-generation permeation enhancers, region-specific delivery strategies, and quantitative models of epithelial recovery and variability, rather than pursuing oral delivery as a binary, “all-or-nothing” target [345,478].

Beyond antibodies and metabolic peptides, the broader delivery landscape is being reshaped by advances in long-acting depots, sustained-release formulations, and route diversification, recognizing pharmacokinetic optimization and half-life extension as a multiparameter problem. While molecular engineering strategies such as lipidation and Fc/albumin fusion are well established, their application is becoming more nuanced, with increasing attention paid to target-mediated drug disposition, tissue selectivity, and peak-to-trough exposure control [39,40,97,98,100,114–118,261]. Despite their nuances, lipidation/albumin-binding strategies continue to mature, supported by mechanistic understanding of albumin hitchhiking and its impact on exposure profiles and dosing intervals [56,97–100].

#### 6.1.4. Lipid nanoparticles

LNPs have achieved industrial-scale non-viral delivery, and the field is now translating those manufacturing and regulatory learnings into intracellular delivery of proteins and protein-like payloads. Recent works demonstrate that LNP designs can deliver diverse proteins into living cells while retaining function, demonstrating the maturation of LNP engineering and tuning for endosomal escape and cargo compatibility from early “encapsulate and hope” approaches [220,221,225–227]. However, LNP success has also exposed a key truth: in vivo performance is dominated by complex nano–bio interactions that are still incompletely predictable. For instance, recent studies show how protein coronas formed in vivo can redefine nanoparticle identity and compromise delivery outcomes—an effect that complicates translation and points toward the need for better predictive assays, standardized corona characterization, and next-generation stealth/biomimetic surfaces [173,218,222,479]. Alternative administration strategies such as pulmonary, transdermal, and microneedle-based delivery are advancing in focused indications where local exposure, needle avoidance, or rapid onset offer clear advantages [365–373,376–394].

#### 6.1.5. Administration strategies

Microneedle patches have evolved from vaccine-focused concepts to broader protein/peptide delivery platforms, with innovations in dissolving and coated architectures aimed at improving stability, reducing cold-chain reliance, and enabling painless self-administration [387,388]. However, the field must still address practical scaling constraints including dose loading, patch size limits, and reproducible insertion across skin types before microneedles can displace injections for higher-dose biologics at scale [371–373,376–386]. In the pulmonary space, renewed interest in inhaled biologics is driven by improved particle engineering, devices, and the attractiveness of local delivery for respiratory disease. Systemic pulmonary delivery remains viable for select indications, with insulin as the historical anchor, but broader adoption will hinge on long-term safety monitoring and clear advantages over SC regimens, especially in chronic diseases [280,286–290].

#### 6.1.6. Artificial intelligence

Artificial intelligence (AI) is increasingly influencing the discovery and development of peptide and protein therapeutics, although its current value is best understood as augmentative rather than autonomous. Recent reviews emphasize that AI now spans nearly the full pharmaceutical pipeline, including target identification, hit discovery, structure generation, property prediction, retrosynthetic planning, biomarker discovery, and clinical-trial design [480–484]. However, performance remains highly dependent on data quality, benchmark design, and prospective experimental validation rather than on algorithmic novelty alone. AI is no longer a peripheral computational aid, but it has not yet eliminated the need for rigorous biophysical characterization, medicinal chemistry, formulation science, and translational pharmacology.

For peptide and protein therapeutics, AI is especially well positioned because these modalities are intrinsically information rich. Sequence-based language models, structure-prediction systems, and geometric deep-learning frameworks can now help prioritize mutations, forecast folding and binding interfaces, estimate developability risks, and narrow experimental search space during antibody, enzyme, and peptide engineering [480–484]. This is particularly relevant to the delivery of therapeutic peptides and proteins, as delivery performance often depends not only on the carrier but also on cargo properties and membrane interaction. AI therefore offers a route to more integrated design, in which molecular engineering and delivery constraints are considered simultaneously rather than sequentially. Recent reviews collectively suggest that the most durable value of AI has emerged not from “one-shot” de novo design claims, but from iterative, closed loops of design, development, delivery, and analysis [480–484].

Machine learning is being used to accelerate formulation development, reduce empirical screening burdens, and identify nonlinear relationships among excipient composition, process parameters, particle size, encapsulation efficiency, release kinetics, and biological performance [485–488]. These approaches are particularly attractive for complex systems such as lipid nanoparticles, long-acting injectables, and oral formulations, where formulation space is high-dimensional and experimentally expensive to explore exhaustively [485–488]. Rather than replacing experimental formulation science, AI is helping to prioritize experiments, guide multi-objective optimization, and support “computational pharmaceutics” workflows that integrate molecular descriptors, process variables, and pharmacokinetic targets.

Within peptide and protein delivery specifically, the near-term opportunity for AI is likely to be greatest in formulation optimization and developability prediction, rather than in fully autonomous therapeutic invention. For example, AI-guided workflows are increasingly being applied to predict which cargos are compatible with specific carrier chemistries, to optimize ionizable or helper lipid selection for LNPs, to estimate nanoparticle biodistribution, and to connect in vitro release or transport data with in vivo exposure models [487–490]. This is a meaningful shift for the field: Many longstanding barriers in peptide and protein delivery arise from multivariate interactions among cargo structure, excipient identity, particle architecture, route of administration, and biological environment.

However, the present status of AI in this area should not be overstated. The major bottlenecks are now data-centric: sparse and non-standardized formulation datasets, inconsistent reporting of failed experiments, weak interoperability across laboratories, limited external validation, and insufficient integration of mechanistic biophysics with machine learning [485–487], As a result, models trained on narrow datasets may generalize poorly across cargo classes, carrier platforms, or administration routes.

Looking forward, AI is likely to be most transformative where it helps unify currently fragmented design decisions across molecular engineering, formulation, manufacturing, and translation [484–487]. For peptide and protein therapeutics, this could include joint optimization of sequence and carrier selection, prediction of manufacturability and storage stability, rational matching of delivery route to cargo properties, and patient-stratified selection of nanomedicine or biologic regimens. In this sense, AI should be viewed not as a separate technological trend, but as an enabling layer that may increasingly connect the major themes of this review: structural optimization of biologics, carrier design, and route-specific delivery strategy [484–487]. The central challenge for the next few years will be to convert promising computational performance into experimentally validated, reproducible, and clinically relevant advances in peptide and protein drug delivery.

### 6.2. Key scientific and translational challenges for the next decade

#### 6.2.1. Intracellular delivery and the endosomal escape barrier

One of the most fundamental scientific challenges in peptide and protein therapeutics is achieving efficient intracellular delivery of intact macromolecules. Although biologic drugs have achieved widespread clinical success against extracellular targets, most intracellular protein targets remain inaccessible because folded proteins generally cannot cross cellular membranes without assistance. Endosomal sequestration remains a dominant barrier to functional delivery with nanocarrier platforms [171,172]. Quantitative studies consistently demonstrate that only a small fraction of internalized cargo escapes from endosomal compartments into the cytosol, where therapeutic proteins must operate to modulate intracellular signaling or gene editing pathways [491,492]. Indeed, experimental measurements of nanoparticle-mediated delivery often report cytosolic release efficiencies below 10%, with most internalized cargo undergoing lysosomal degradation or recycling back to the extracellular space [171–173,491,493]. This limitation has been widely recognized as a major rate-limiting step in nanomedicine and nucleic acid delivery systems, including LNP platforms used for mRNA therapeutics and vaccines [43–45,160–168,216,491,493]. Addressing this barrier will require deeper mechanistic understanding of endosomal membrane destabilization, nanoparticle phase behavior, and intracellular trafficking dynamics. Emerging strategies to address cytosolic delivery include pH-responsive lipids [220,221], fusogenic peptides [191], biomimetic membrane fusion systems [494], and pathogen-inspired escape mechanisms. Continued progress in this area will be critical for enabling intracellular protein therapeutics, gene-editing ribonucleoprotein complexes, and other next-generation biologic modalities.

#### 6.2.2. Overcoming biological barriers to non-parenteral delivery

Despite decades of investigation, enabling reliable non-parenteral delivery of peptides and proteins remains a central translational challenge. Biological barriers such as proteolytic degradation, epithelial tight junctions, mucus layers, and rapid clearance limit systemic absorption of macromolecules across mucosal surfaces such as the gastrointestinal tract, lung, and nasal epithelium [252,296]. Oral peptide delivery in particular has long been described as the “holy grail” of biologic drug development because of its potential to improve patient compliance and reduce healthcare burden. However, the clinical success of oral semaglutide illustrates that effective solutions are unlikely to arise from universal nanoparticle platforms alone. Instead, successful strategies increasingly rely on localized, mechanism-specific approaches that transiently modify epithelial barrier properties while protecting the peptide from enzymatic degradation [33,495]. Absorption enhancers such as SNAC (Fig. 5C) exemplify this paradigm by promoting localized epithelial permeability and stabilizing peptides within the gastric microenvironment, thereby enabling clinically meaningful bioavailability for highly potent peptides [345,495,496]. Nevertheless, the broader application of permeation enhancers remains constrained by safety considerations, including epithelial irritation, long-term barrier disruption, and variability in gastrointestinal physiology among patients.

#### 6.2.3. Integrating molecular engineering with delivery system design

Historically, peptide and protein therapeutics were often optimized for biological potency before delivery feasibility was considered during later formulation stages. Increasingly, however, clinical experience suggests that successful biologics require simultaneous optimization of molecular design and delivery compatibility. The development of high-concentration antibody formulations provides a clear example of this shift. Subcutaneous administration of monoclonal antibodies frequently requires concentrations exceeding 100–200 mg/mL, where viscosity, protein-protein interactions, and aggregation propensity become critical formulation challenges [281,282,286,497]. Consequently, antibody engineering strategies now routinely incorporate modifications that influence colloidal stability, surface charge distribution, and isoelectric point during early drug discovery to ensure compatibility with high-concentration delivery formats [39,40,61, 106,125,131,144,147–150,153–158]. Similar integration is emerging in other protein and peptide therapeutics, where PEGylation, lipidation, albumin-binding motifs, backbone stabilization, and Fc-fusion strategies are incorporated into peptide scaffolds to enable long-acting pharmacokinetics and sustained-release formulations [56,65,69,91,93,105,111,114–121]. Advances in structural biology, computational protein design, and machine learning–guided sequence optimization are accelerating this convergence between molecular engineering and formulation science. Tools such as deep-learning–based structural prediction and generative protein design algorithms now enable rational exploration of sequence space to identify variants that simultaneously optimize potency, stability, and developability [133–139,498]. In the future, integrating these computational approaches with high-throughput formulation screening may enable predictive design workflows in which delivery constraints are incorporated directly into early therapeutic design.

#### 6.2.4. Manufacturing scalability and chemistry, manufacturing and controls

While numerous delivery platforms demonstrate promising results in preclinical models, translation into approved therapeutics is frequently limited by manufacturing complexity and regulatory constraints. Nanoparticle formulations, depot systems, and conjugated biologics often require precise control over particle size distribution, encapsulation efficiency, release kinetics, and physicochemical stability [48–51,173,180,181,222]. Variability in these parameters can significantly influence pharmacokinetics, biodistribution, and immunogenicity, making reproducible large-scale manufacturing a major barrier to clinical translation [48–51,173,222]. For example, nanoparticle systems such as liposomes and LNPs require highly controlled mixing processes to ensure uniform particle formation and consistent encapsulation properties [160–168]. Recent advances in microfluidic manufacturing technologies and continuous-flow nanoparticle synthesis have significantly improved process reproducibility, enabling scalable production of clinically validated LNP formulations for nucleic acid therapeutics [238,239]. In parallel, advances in analytical characterization—including cryo-electron microscopy, small-angle scattering, and advanced chromatographic techniques—are improving the ability to monitor nanoparticle structure and stability during manufacturing. Nevertheless, regulatory frameworks for complex nanomedicines remain evolving, and early integration of CMC considerations during therapeutic discovery will likely become increasingly important for successful translation. Future progress in biologic delivery therefore depends not only on advances in carrier design but also on innovations in scalable manufacturing, real-time process monitoring, and regulatory science.

#### 6.2.5. Improving predictive translational models

A final critical challenge in macromolecular drug delivery is the limited predictive power of current preclinical models. Many delivery systems that demonstrate strong efficacy in rodent models ultimately fail in clinical development due to differences in physiology, immune responses, and pharmacokinetics between animal models and humans. These translational discrepancies are particularly pronounced for nanoparticle systems, where factors such as protein corona formation, tissue distribution, and immune recognition can differ substantially between species [173,218,222,499–501]. Addressing this gap will require the development of more predictive experimental models capable of recapitulating human biological barriers. Emerging technologies, including organ-on-chip microphysiological systems, advanced humanized animal models, and quantitative pharmacokinetic–pharmacodynamic modeling, offer promising approaches to bridge this translational divide [502,503]. By combining these systems with advanced imaging and systems biology approaches, researchers may be able to more accurately predict the behavior of delivery systems in human tissues before clinical testing. Ultimately, improving predictive translational models will be essential to reduce development risk, accelerate clinical translation, and guide the rational design of next-generation peptide and protein delivery platforms.

### 6.3. Outlook

Collectively, these challenges suggest that the future of peptide and protein therapeutics will depend less on any single technological breakthrough and more on integrated delivery engineering that simultaneously addresses molecular design, formulation stability, device compatibility, manufacturability, and patient usability. The most successful therapies of the past decade, from long-acting incretin analogs to high-concentration antibody formulations, have emerged from coordinated innovation across these domains rather than isolated advances in any one discipline. Looking forward, continued progress will likely arise from convergence between molecular engineering, delivery science, and quantitative modeling.

## Figures and Tables

**Table 1:** Top ten peptide and protein therapeutics by global sales in 2024. Ozempic^®^ and Mounjaro^®^ are proteins, while the rest of the drugs in this table are peptides. [15,16].

Rank	Drug Name	Disease Area	Manufacturer(s)	2024 Sales (Million USD)	Growth Rate 2023–2024 (%)
1	Keytruda^®^: (pembrolizumab)	Oncology	Merck	29,482.00	17.9
2	Ozempic^®^: (semaglutide)	Metabolic Diseases	Novo Nordisk	17,451.00	25.8
3	Dupixent^®^ (dupilumab)	Immunology/Respiratory	Sanofi/Regeneron	14,147.00	22.1
4	Skyrizi^®^ (risankizumab-rzaa)	Immunology	AbbVie	11,718.00	50.9
5	Darzalex^®^ (daratumumab) & Darzalex Faspro^®^ (daratumumab and hyaluronidase-fihj)	Oncology/Hematology	Johnson & Johnson	11,670.00	19.8
6	Mounjaro^®^: (tirzepatide)	Metabolic Diseases	Eli Lilly and Company	11,540.10	123.5
7	Stelara^®^: (ustekinumab)	Immunology	Johnson & Johnson	10,361.00	−4.6
8	Eylea^®^: (aflibercept)	Ophthalmology	Regeneron/Bayer	9546.00	−25.9
9	Opdivo^®^: (nivolumab)	Oncology	Bristol-Myers Squibb	9304.00	3.3
10	Humira^®^: (adalimumab)	Immunology	AbbVie	8993.00	−37.6

**Table 2:** Comparative strategies to improve stability and bioactivity in peptide and protein therapeutics. Information compiled from in-text references from Subsection 5.2, with rankings (low-very high) assigned accordingly at the judgement of the authors.

Strategy	Primary Mechanism	Effect on Proteolysis	Effect on Bioactivity	Structural Disruption	Clinical Maturity	Key Limitations
Backbone Modifications (D-aa, *N*-methylation, noncanonical residues)	Blocks protease recognition; increases rigidity	High	Usually preserved; can enhance selectivity	Moderate	High	May reduce solubility or alter folding
Side-Chain Modifications	Enhances stability, solubility, or affinity	Moderate	Often improved	Low–Moderate	High	Risk of aggregation or altered receptor binding
N-/C-Terminal Modifications	Blocks exopeptidase cleavage	Moderate	Minimal direct enhancement	Low	Very High	Limited protection from endopeptidases
Cyclization	Eliminates termini; conformational constraint	High	Often increased affinity	Moderate	Increasing	Synthetic complexity
Peptoids	Backbone reconfiguration	Very High	Requires redesign	High	Preclinical	Conformational unpredictability

**Table 3:** Comparative strategies to improve pharmacokinetics and pharmacodynamics for peptide and protein therapeutics. Information compiled from in-text references from Subsection 5.3, with rankings (low-very high) assigned accordingly at the judgement of the authors.

Strategy	PK Effect	PD Modulation	Immunogenic Risk	Clinical Maturity	Key Limitations
PEGylation	↑ Half-life via hydrodynamic shielding and reduced renal filtration	Minimal direct PD change	Anti-PEG antibodies possible	High	Reduced receptor binding near active sites; potential PEG accumulation; accelerated blood clearance due to anti-PEG antibodies
Lipidation	↑ Half-life via reversible albumin binding	Usually minimal; may alter receptor kinetics if near binding domain	Low–Moderate	Very High	Risk of aggregation; altered biodistribution if excessive hydrophobicity; formulation complexity
Glycosylation/Glycoengineering	↑ Stability; modulates FcRn recycling; reduced clearance	Can enhance effector functions	Low	Very High	Glycoform heterogeneity; manufacturing complexity; species-specific glycosylation differences
Fc/Albumin Fusion	Dramatic half-life extension via FcRn recycling	May alter signaling valency or receptor clustering	Low–Moderate	Very High	Increased molecular size may limit tissue penetration; altered pharmacodynamics due to dimerization; complex chemistry, manufacturing, and control considerations
Peptide–Drug Conjugates (PDCs)	Variable; can be extended via albumin-binding motifs	Strong spatial PD control via cleavable linkers	Low	Growing	Linker instability or premature cleavage; off-target toxicity; complex optimization of targeting versus payload release

**Table 4:** Comparative strategies to improve targeting and potency. Information compiled from in-text references in Subsection 5.4, with rankings (low-very high) assigned accordingly at the judgement of the authors.

Strategy	Specificity	Penetration	Engineering Complexity	Potency Enhancement	Clinical Maturity	Key Limitations
Directed Evolution	Indirect (affinity maturation)	N/A	High	High	Very High	Screening burden
Rational Design	Indirect	N/A	Moderate	Moderate–High	Very High	Requires structural data
CPPs	Low–Moderate	High (intracellular)	Low	Moderate	Low-Moderate	Off-target uptake
CTPs	Moderate–High	Moderate	Moderate	Moderate	Moderate-High	Receptor heterogeneity
ADCs	Very High	Moderate	Very High	Very High	Very High	Heterogeneity, toxicity

**Table 5:** Comparative drug carrier platforms. Information compiled from in-text references from Section 6, with rankings (low-very high) assigned accordingly at the judgement of the authors.

Carrier	Protection from Proteolysis	Release Control	Systemic Delivery	Clinical Maturity	Key Limitations
Micelles	Moderate	Limited	Possible	Moderate	Instability upon dilution
Liposomes	High	Moderate	Yes	High (non-peptide)	Leakage, cost
Emulsions	Low–Moderate	Limited	Oral/topical	High	Surfactant stress
Hydrogels	High (local)	Excellent	Mostly local	Very High	Limited systemic use
LNPs	High	Moderate	Yes	Very High	Encapsulation efficiency
SLNs/NLCs	Moderate	Moderate	Oral/mucosal	Low	Polymorphism, stability
Polymeric NPs	High	Tunable	Yes	High (vaccines)	Manufacturing reproducibility

**Table 6:** Select parenteral peptide and protein therapeutics. Selection is curated to highlight seminal, prominent, or commercially successful drugs in the US market.

Brand Name (generic name)	Primary Indication: IV, SC, IM or IT	Drug Size: Composition	Formulation and Administration Technologies: Key Delivery Challenge(s) Addressed	Year of FDA-Approval or Trial Initiation	Reference (s)
Activase^®^ (alteplase)	Acute ischemic stroke, pulmonary embolism, acute myocardial infarction: IV	527 aa: Recombinant human form of tissue plasminogen activator	Lyophilized powder (to be reconstituted)	IV: 1987	[255–258]
TNKase^®^ (tenectaplase)	Acute ischemic stroke, pulmonary embolism, acute myocardial infarction: IV	527 aa: Recombinant human form of tissue plasminogen activator with mutations introduced to increase fibrin specificity and glycosylation	Lyophilized powder (to be reconstituted): Glycosylation to increase half-life	IV: 2000	[259,260]
Ozempic^®^, Wegovy^®^ (semaglutide)	T2DM (Ozempic^®^), weight management (Wegovy^®^): SC	31 aa: Glucagon-like peptide-1 receptor agonist with addition of PEG moiety to promote albumin binding and increase half life	Solution (pre-filled pen): Lipidation to protect against proteolytic degradation	SC: 2017	[21,22,31,33,57,58]
Victoza^®^, Saxenda^®^ (liraglutide)	T2DM (Victoza^®^), weight management (Saxenda^®^): SC	31 aa: Glucagon-like peptide-1 receptor agonist with addition of palmitic acid to promote albumin binding and extend half-life	Solution (pre-filled pen): Lipidation to protect against proteolytic degradation	SC: 2010	[30,56,261]
Mounjaro^®^, Zepbound^®^ (tirzepatide)	T2DM (Mounjaro^®^), weight management (Zepbound^®^): SC	39 aa: Dual glucagon-like peptide-1 agonist and glucose-dependent insulinotropic polypeptide with addition of eicosanedioic acid to promote albumin binding and resistance to enzymatic degradation	Solution Mounaro^®^: pre-filled pen Zepbound^®^: pre-filled pen or single-dose vial: Lipidation to protect against proteolytic degradation	SC: 2022	[262,263]
Herceptin^®^, Herceptin Hylecta^®^ (trastuzumab)	HER2-positive breast cancer, metastatic gastric cancer (Herceptin^®^): SC	1328 aa	Solution administered in conjunction with rHuPH20: RHuPH20 allows for larger injection volumes to improve patient compliance	IV: 1998 SC: 2019	[264–266]
Enflonsia^®^ (clesrovimab)	RSV prevention in infants: IM	1312 aa: Fc aa substitutions to enhance stability in acidic environments	Solution (pre-filled syringe): Fc fusion for enhanced targeting and half life	IM: 2025	[267–269]
Prialt^®^ (ziconotide)	Pain management: IT	25 aa	Isotonic solution; administered using microinfusion device: Intrathecal administration bypasses the blood-brain barrier	IV: 2004	[254,270–273]

**Table 7:** Orally delivered peptides and proteins FDA-approved (except for 3D). (A) Selected Classical orally delivered peptides and proteins; (B) Oral proteins used for replacement of enzymes in gut; (C) Oral peptides/peptidomimetics with alternative classification; (D) Select peptides/proteins or technologies in the pipeline for oral use.

Brand Name (generic name): Dosage Form	Primary Indication: Local or Systemic Use	Drug Size: Composition	Formulation and Administration Technologies: *Key Delivery Challenge(s) Addressed*	Year of FDA-Approval or Trial Initiation	Reference (s)
A. Select Classical Orally Delivered Peptides and Proteins					
Sandimmune^®^, Neoral^®^ (cyclosporine): Oral liquid, soft gel capsule	Immunosuppressant: Systemic use	11 aa: Cyclic peptide	Sandimmune^®^ is an oil-based oral solution (liquid and capsule dosage forms). Neoral^®^ oral liquid forms a microemulsion in aqueous fluids: *Improves bioavailability using SMEDDS (Self-Microemulsifying Drug Delivery Systems) technology*	Sandimmune^®^ Liquid: 1983 Capsules: 1990 Neoral^®^: 1995	[59,197,321,322]
Lupkynis^®^ (Voclosporin): Capsule	Immunosuppressant mainly for treatment of lupus nephritis: Systemic use	11 aa: Cyclosporine analogue with a carbon extension on the 1st amino acid	No special delivery technologies since its structural modification increases potency (binding to cyclophilin A) and reduces degradation in gut	2021	[336]
Desmotabs^®^ (desmopressin acetate, or DDAVP): Oral tablet	Oral tablets for diabetes insipidus, SL tablets for nocturnal polyuria: Systemic use	9 aa: Synthetic vasopressin analog with modified cysteine	Oral tablet uses standard tablet excipients such as disintegrants: *Enhances absorption in small intestine, and drug is inherently more stable than other peptides due to chemical modification and size*	DDAVP Oral tablets: 1995	[337–342]
Mycapssa^®^ (octreotide): Capsule	Somatostatin analog for acromegaly: Systemic use	8 aa: Cyclic peptide with disulfide bridge, two D-amino acids, and modified C-terminus	Uses TPE^®^ C8 permeation enhancer technology (Fig. 5D) for oily suspension formulation inside hard capsule plus Acryl-EZE^®^ enteric coating: *TPE may expand tight junctions (as in* Fig. 5B*) for paracellular transport between epithelial cells in small intestine; may also have transcellular mechanisms*	1988 Capsule: 2020	[332,333,343–345]
Rybelsus^®^ (semaglutide): Tablet	GLP-1 agonist for treatment of type 2 diabetes: Systemic use	31 aa: (94% homology with GLP-1) with modifications to improve affinity to albumin in and proteolytic resistance	Co-formulated with absorption enhancer SNAC (sodium N-[8 (2-hydroxylbenzoyl) amino] caprylate): *Improves absorption in stomach, protects from enzymatic degradation, and improves drug solubility*	Ozempic^®^: 2017 Rybelsus^®^: 2019	[33,36]
Palforzia^®^ (peanut allergen powder): Powder-filled capsule, powder (sachets)	Peanut allergy: Systemic use	Mixture of 4 peanut protein allergens with MW between 15 and 64 kDa (for monomers)	No special formulation besides typical excipients used in tablet/capsules for capsule; powdered sachet has no special formulation.	Ages 4–17: 2020 Ages 1–3: 2024	[346–349]
B. Example of Oral Protein used for Replacement of Enzymes in Gut					
Sucraid^®^ (sacrosidase): Oral solution	Congenital sucrase-isomaltase deficiency (CSID): Local use	513 aa as monomer, but may also exist in dimer, tetramer, and octamer forms	No special formulation since site of action is small intestine as an oral replacement therapy; acid-labile and should be taken with food/milk	1998	[350,351]
C. Oral Peptides/Peptidomimetics with Alternative Classification					
Vancomycin: Oral solution, capsules	*C. difficile* infection in intestinal lumen (not systemically absorbed): Local use	7 aa: Tricyclic glycopeptide, classified as an antimicrobial	Vancomycin is small and inherently protease resistant due to its structure	1958	[352–354]
Macrilen^®^ (macimorelin): Granules (sachet) to be reconstituted in water	Diagnosis of adult growth hormone deficiency, GHS-R1a agonist: Systemic use	Tripeptide-derived peptidomimetic (not a true peptide/protein)	Peptidomimetic (small molecule) with non-natural components (α-aminoisobutyric acid and a D-tryptophan derivative): *Improves proteolytic resistance after transmucosal absorption in the small intestine*	2017	[355]
D. Select Peptides/Proteins or Technologies in the Pipeline for Oral Use					
MK-0616: Capsules	PCKS9 inhibitor, high cholesterol; Systemic use	13 aa; Macrocycle with multiple modifications	Lead compound found using mRNA display technology, with further refinement of chemistry: *Minimizes oxidation susceptibility while maintaining potency, increasing solubility, and minimizing isomer formation*	Completed Phase 2b clinical trials in 2023; now in Phase 3	[356–358]
Tregopil (IN-105): Tablet	Type 1 diabetes: Systemic use	51 aa; PEGylated human recombinant insulin	Methoxy polyethylene glycol derivative attached to B29 lysine enhances its stability: sodium caprate used as a permeation enhancer to increase GI absorption	Completed Phase 2/3 clinical trials in US in 2020; no further updates available	[359–362]
RaniPill^®^ robotic pill for peptide and protein delivery: Capsule	Various: Systemic use via transenteric delivery	Tested for delivery of various antibodies and other biotherapeutics	Enteric coated oral robotic pill that dissolves in the intestine, resulting in mixing of citric acid and sodium bicarbonate to liberate CO2 gas: *Gas inflates a balloon that aligns a microsyringe to auto-inject the biotherapeutic into the intestinal wall (with the capsule contents excreted in the feces, and needle dissolving within 15 mm)*	*As of* 2025, several products in Phase 1–2 clinical trials.	[363,364]

**Table 8:** Peptide and protein therapeutics (A) approved for topical use by the FDA, (B) in the research pipeline for topical use, and (C) in the research pipeline for transdermal use.

Brand Name (generic name): Dosage Form	Primary Indication: Local or Systemic Use	Drug Size: Composition	Formulation and Administration Technologies: *Key Delivery Challenge(s) Addressed*	Year of FDA-Approval or Trial Initiation	Reference (s)
A. FDA-approved Topical Peptides and Proteins					
Regranex^®^ (Becaplermin): Topical gel	Diabetic neuropathic foot ulcers: Local use	109 aa	Hydrogel-based topical gel for *sustained local delivery: Allows stable, localized delivery of growth factor and avoids systemic exposure*	1997	[374,375]
Recothrom^®^ (thrombin topical (recombinant): Lyophilized powder	Hemostasis during surgery: Local use	295 aa: Dimer with a disulfide linkage between chain A (36 aa) and chain B (259 aa)	Comes as a lyophilized powder that is reconstituted in a supplied diluent: *Allows controlled local clot formation*	2008	[395,396]
Raplixa^®^ (fibrin sealant (human)): Topical powder	Adjunct to hemostasis for mild to moderate bleeding during surgery: Local use	Fibrinogen and Thrombin	Spray-dried, lyophilized human fibrinogen and thrombin blended into dry powder, applied with or without RaplixaSpray^™^	2015	[397]
Vistaseal^®^ (fibrin sealant (human)): Topical solution kit	Adjunct to hemostasis when conventional methods are ineffective or impractical: Local use	Fibrinogen (~340 kDa) and Thrombin (~36 kDa)	Two-component frozen liquid (thrombin + fibrinogen), dual-syringe applicator with drip or airless spray delivery	2017	[398]
B. Investigational Peptides and Proteins for Topical Use					
Viaskin Peanut: Epicutaneous immunotherapy patch	Peanut allergy in toddlers aged 1–3 years: Local Use	Dry peanut allergen	Epicutaneous delivery system with lyophilized peanut protein in an occlusive patch chamber: *Allows needle-free immunotherapy and controlled allergen exposure to the superficial layers of the skin, and avoids direct contact with the circulatory system, which reduces the risk of severe systemic reactions*	2023 Phase 3 clinical trial	[376]
CTB-ACE2: Topical gum	Reduction of SARS-CoV-2 viral load in saliva: Local use	Pentameric fusion protein consisting of CTB and ACE2 ectodomain	TB-ACE2 fusion protein expressed in plant chloroplasts, bioencapsulated in lyophilized plant cells, formulated into chewing gum	2022 Phase 1/2	[399]
C. Investigational Peptides and Proteins for Transdermal Use					
(Novolin^®^) (human regular insulin): Transdermal patch	Management of insulin-dependent diabetes mellitus: Systemic use	~5.8 kDa	Intraepidermal Delivery (IED) using short solid microneedles (150 μm) + Iontophoresis (IP) patch: *Overcomes the stratum comeum and allows controlled basal and on-demand bolus insulin delivery*	2012 In preclinical dev	[387]
Peptide A: Coated microneedle patch	Proprietary: Systemic use	29 aa linear peptide: Palmitoylated lysine	Coated solid microstructure transdermal system (sMTS) composed of polymeric *microneedle arrays* (316 needles per 1.27 cm^2^ patch), applied to needle tips using a proprietary dip-coating process	2020	[400]
Insulin: Transdermal microneedle patch	Diabetes mellitus: Systemic use	51 aa	*Dissolving microneedles* using insulin-loaded CaCO_3_ microparticles and PVP polymer matrix	2018 In preclinical dev	[388]

**Table 9:** FDA-approved peptide and protein therapeutics as delivered by (A) intranasal, (B) pulmonary, (C) sublingual, (D) topical ocular, and (E) intravitreal ocular administration. Abbreviation explanation for Table 5D: AMD: age-related macular degeneration, DME: diabetic macular edema, ROP: retinopathy of Prematurity, nAMD: neovascular age-related macular degeneration, VMA: vitreomacular adhesion, VRI: vitreoretinal interface, DR: diabetic retinopathy, RVO: macular edema following retinal vein occlusion, GA: Geographic atrophy.

Brand Name (generic name): Dosage Form	Primary Indication	Drug Size: Composition	Formulation and Administration Technologies: *Key Delivery Challenge(s) Addressed*	Year of FDA-Approval or Trial Initiation	Reference (s)
A. Intranasal					
Syntocinon^®^ (oxytocin): Solution	Postpartum lactation, delayed or excessive pregnancy, gestational hypertension	9 aa	Aqueous metered-dose solution delivered via spray pump for rapid mucosal absorption: *Noninvasive route that bypasses GI degradation/first-pass metabolism and supports rapid onset of effect*.	1964 (Discontinued in 1997 due to market reasons)	[417,418]
Miacalcin^®^ Nasal, Fortical^®^ (salmon calcitonin): Solution	Postmenopausal osteoporosis	32 aa	Aqueous metered-dose solution delivered via spray pump for rapid mucosal absorption: *Noninvasive route that bypasses GI degradation/first-pass metabolism and supports rapid onset of effect*.	Miacalcin^®^: 1995 Fortical^®^: 2005	[404,419,420]
Baqsimi^®^ (glucagon): Powder	Severe hypoglycemia	29 aa: Human recombinant peptides	Preservative-free intranasal dry powder (single-use device) containing beta-cyclodextrin, dodecylphospho-choline as excipients: *Eliminates reconstitution and injection steps* for regular glucagon, enabling rapid, user-friendly rescue dosing during severe hypoglycemia.	2019	[406,408]
B. Pulmonary					
Pulmozyme^®^ (dornase alfa): Solution	Cystic fibrosis	260 aa: Recombinant human DNase I enzyme	Inhalation solution administered by nebulizer to deliver active enzyme directly to the airways for local action: *High local exposure at the disease site while minimizing systemic exposure and avoids GI degradation*	1993	[421,422]
Exubera^®^ (insulin): Powder	Rapid-acting insulin for Type 1 & 2 diabetes (mealtime control)	Recombinant human insulin	Unit-dose blister dry powder insulin formulation for systemic absorption through the lung: Direct pulmonary delivery enables *high local exposure at the disease site white minimizing systemic exposure and avoids GI degradation*	2006 (Discontinued in 2007)	[423,424]
Afrezza^®^ (insulin): Microparticle	Rapid acting prandial insulin for Type 1 & Type 2 diabetes	50 aa: Recombinant human insulin	Single-use cartridges of inhalation powder administered via Dreamboat inhaler to achieve rapid prandial systemic exposure: Needle-free mealtime insulin delivery with *rapid onset enabled by Technosphere particles (FDKP microparticles) for deposition and fast dissolution/absorption*	2014	[425,426]
C. Sublingual					
Nocdurna^®^ (desmopressin acetate): Tablet	Nocturnal enuresis	9 aa: (1st cysteine is modified), Synthetic analog of vasopressin	SL tabs *avoid first-pass in liver and enzymatic degradation in GI tract*	2018 (Discontin-ued in 2023 due to patent expiration)	[178,342]
Ragwitek^®^ (short ragweed pollen allergen extract): Tablet	Immunotherapy for ragweed pollen-induced allergic rhinitis, systemic use via immune system	38 kDa: Pectate lyase enzyme (Amb a 1 and other allergens)	Standard rapidly dissolving sublingual tablet: *Drug is captured by Langerhans cells and dendritic cells in oral mucosa (not passive diffusion), subsequently inducing immune tolerance*	Ages 18–65: 2014 Ages 5–17: 2021	[427,428]
D. Ocular—Topical					
Restasis^®^ (cyclosporine A): Nanoemulsion	Dry Eye Disease (chronic keratoconjunctivitis sicca with inflammation)	11 aa: Cyclic immunosuppressive	Oil-in-water ophthalmic nanoemulsion to disperse a highly lipophilic cycle peptide for topical dosing: *Iontophoresis (cationic surface charge in newer versions) improves ocular surface delivery of a poorly water-soluble peptide while limiting systemic exposure*	2003	[429,430]
Oxervate^®^ (cenegermin-bkbj): Solution	Neurotrophic keratitis (corneal neurotrophic ulceration, stages 1–3)	118 aa: Human nerve growth factor, neurotrophic peptides	Aqueous ophthalmic solution supplied for topical corneal dosing with frequent administration. *Enables labile proteins to the corneal surface despite tear dilution/rapid clearance, while maintaining stability through controlled handling*	2018	[431–433]
E. Ocular—Intravitreal Injection					
Eylea^®^, Eylea HD^®^ (aflibercept): Solution	wet AMD, DME, ROP, DR	432 aa: Fusion protein of VEGFR-1 and VEGFR-2 extracellular domains and human IgGl Fc	ISO-osmotic aqueous solution of dimeric VEGF “decoy receptor” fusion protein via disulfide bond *VEGF-trap): *Achieves high posterior-segment exposure and sustained VEGF inhibition while minimizing systemic exposure*.	2011	[323,324]
Yesafili^®^ (aflibercept-jbvf): Solution	Wet AMD, EVO, DME, DR	Interchangeable biosimilar to aflibercept	Biosimilar VEGF-trap, interchangeable biosimilar to Eylea HD^®^	2023	[434]
Opuviz^®^ (aflibercept-yszy): Solution	Wet AMD, EVO, DME, DR	Interchangeable biosimilar to aflibercept	Biosimilar VEGF-trap, interchangeable biosimilar to Eylea HD^®^	2024	[434]
Avastin^®^ (bevacizumab): Solution	FDA-approved (IV): systemic cancer Off-label (Intravitreal injection): wet AMD, DR, DME	Humanized IgGl mAb targeting VEGF-A	Recombinant mAb via CHO cells: Intravitreal use typically requires aseptic repackaging into small-volume syringes.	2004	[435]
Beovu^®^ (brolucizumab-dbll): Solution	nAMD, DR, DME, wet AMD	26 aa: Single-chain anti-VEGF humanized scFv fragment	Nanobody-type single-chain mAb fragment targeting VEGF-A; recombinant expressed in *E. coli:* Smaller biologic format enables *high molar dosing in a small volume to improve tissue penetration of VEGF blockade*.	2019	[436,437]
Lucentis^®^ (ranibizumab): Solution	DME, DR, wet AMD	Recombinant humanized IgG1 kappa isotype monoclonal antibody	Recombinant Fab produced in *E. coli*, no special delivery technologies.	2006	[438,439]
Byooviz^®^, SB11 (ranibizumab-nuna): Solution	Wet AMD, DME, RVO	Biosimilar of ranibizumab	Recombinant Fab produced in *E. coli* biosimilar to Lucentis^®^	2021	[440–442]
Susvimo^®^ (ranibizumab-swnb): Solution	Wet AMD	115 aa: Ranibizumab fragment, implant form	Refillable ocular implant (port delivery system) that continuously releases ranibizumab into the vitreous with periodic in-office refills: Sustained intraocular delivery reduces injection frequency and treatment burden compared with repeated bonus intravitreal injections.	2021	[443–446]
Vabysmo^®^ (faricimab): Solution	nAMD, DME	Bispecific IgG (anti-VEGF-A & Ang-2)	Bispecific antibody targeting VEGF-A and Ang-2 (CrossMAb-engineered format): Dual pathway inhibition with intravitreal delivery aims to improve durability and reduce dosing frequency.	2022	[447,448]
Jetrea^®^ (ocriplamin): Solution	VMA, VRI	Recombinant human plasmin	Recombinant protease intended to cleave vitreoretinal adhesion proteins: Pharmacologic vitreous is provides a *non-surgical approach to release vitreomacular traction/adhesion*	2012	[449]
Syfovre^®^ (pegcetacoplan): Solution	AMD, GA	PEGylated complement C3 inhibitor	PEGylated peptide complement C3 inhibitor designed for ocular complement modulation: *PEGylation supports extended intraocular residence and sustained local target engagement in the posterior segment*	2023	[450,451]

**Table 10:** Comparative delivery strategies by dominant routes of administration. Information compiled from in-text references from Section 7, with rankings (low-very high) assigned accordingly at the judgement of the authors.

Delivery Route	Bioavailability	Patient Compliance	Primary Biological Barrier(s)	Best Use Case	Clinical Maturity	Key Limitations
Intravenous (IV)	Complete	Low	None (direct systemic access)	Acute care biologics; oncology antibodies; enzyme replacement	Very High	Clinical administration required; poor convenience
Subcutaneous (SC)	High	High	Extracellular matrix diffusion; lymphatic uptake	Chronic biologics; monoclonal antibodies; long-acting peptides	Very High	Injection volume limits; site reactions
Intramuscular (IM)	High	Moderate	Muscle diffusion; vascular uptake	Vaccines; depot peptide formulations	Very High	Injection pain; variable absorption
Oral	Very Low	Very High	Gastric acidity; proteases; mucus barrier; epithelial tight junctions; first-pass metabolism	Chronic metabolic peptides; endocrine therapies	Moderate (emerging)	Extremely low bioavailability; formulation complexity
Topical / Transdermal	Very Low (systemic)	Very High	Stratum comeum	Local dermatologic therapy; localized peptide delivery	High (local)	Poor macromolecular permeability
Intranasal	Low-Moderate	High	Mucus barrier; mucociliary clearance; enzymatic degradation	Rapid systemic peptides; CNS-targeting peptides	Moderate	Limited dosing volume; rapid clearance
Pulmonary (Inhalation)	Moderate	Moderate	Airway mucus; alveolar macrophage clearance	Rapid systemic peptides; respiratory diseases	Moderate	Device dependence; dosing variability
Sublingual / Buccal	Low	High	Oral mucosal permeability; salivary dilution	Rapid-acting peptides; rescue therapies	Low-Moderate	Small absorption area; dilution effects
Ocular	Local	Moderate	Tear turnover; corneal epithelium; blood–retinal barrier	Retinal biologics; ophthalmic proteins	Very High	Poor posterior segment penetration; invasive injections

## Data Availability

No data was used for the research described in the article.
